# Body Shape and Alzheimer’s Disease: A Mendelian Randomization Analysis

**DOI:** 10.3389/fnins.2019.01084

**Published:** 2019-10-10

**Authors:** Yuchang Zhou, Xiubin Sun, Maigeng Zhou

**Affiliations:** ^1^Department of Biostatistics, School of Public Health, Shandong University, Jinan, China; ^2^National Center for Chronic and Noncommunicable Disease Control and Prevention, Chinese Center for Disease Control and Prevention, Beijing, China

**Keywords:** Alzheimer’s disease, body shape, Mendelian randomization, survivor bias, simulation analysis

## Abstract

Obesity has been reported to be related to memory impairment and decline in cognitive function, possibly further leading to the development of Alzheimer’s disease (AD). However, observational studies revealed both negative and positive associations between body shape (BS) and AD, thereby making it difficult to confirm causality due to residual confounds and reverse causation. Thus, using genome-wide association study summary data, two-sample Mendelian randomization (MR) analyses were applied to identify whether there exists a causal association between BS and AD. BS was measured using anthropometric traits (ATs) in this study, including body mass index (BMI), waist-to-hip ratio (WHR), waist-to-hip ratio adjusted by body mass index (WHRadjBMI), and waist circumference (WC). The associations of single nucleotide polymorphisms (SNP) with each AT and AD were obtained separately from aggregated data from the Genetic Investigation of Anthropometric Traits (GIANT) consortium and International Genomics of Alzheimer’s Project (IGAP) summary data (17,008 cases with AD and 37,154 controls). An inverse-variance weighted method was applied to obtain the overall causal estimate for multiple instrumental SNPs. The odds ratio (OR) [95% confidence interval (CI)] for AD risk per 1-SD difference in BMI was 1.04 (0.88, 1.23), in WHR was 1.01 (0.77, 1.33), in WHRadjBMI was 1.12 (0.89, 1.41), and in WC was 1.02 (0.82, 1.27). Furthermore, simulation analyses of survivor bias indicated the overall causal effect of BMI on risk of AD was not biased. In conclusion, the evidence from MR analyses showed no casual effect of BS on AD risk, which is inconsistent with the results from previous observational studies. The biological mechanism underlying the findings warrants further study.

## Introduction

Alzheimer’s disease (AD) is the most common form of dementia worldwide in older individuals, accounting for 60–80% of cases (Mathys et al., 2017). It is widely reported that body shape (BS), generally acknowledged to be measured and reflected by anthropometric traits (ATs) such as body mass index (BMI), waist circumference (WC), waist-to-hip ratio (WHR), and waist-to-hip ratio adjusted for body mass index (WHRadjBMI), is associated with the development of several diseases (Winkler et al., 2015; Noyce et al., 2017).

Although there have been many conventional observational studies probing the influence of BS on the risk of AD, the reported results have been controversial. A meta-analysis based on prior systematic reviews provided a pooled relative risk estimate of 1.60 [95% confidence interval (CI): 1.34, 1.92] for BMI with AD, which was confirmed in a more recent meta-analysis of cohort studies (Barnes and Yaffe, 2011; Loef and Walach, 2013). However, another prospective population-based study indicated that each unit increase in BMI was associated with an 8% decreased risk of dementia (Dahl et al., 2008). A case-control study concluded that WC in older adults was significantly lower in the AD group than in the healthy cognition group, inconsistent with the findings of two cohort studies and another cross-sectional study (Luchsinger et al., 2007, 2012; Chu et al., 2009; Habes et al., 2016). Nevertheless, conventional studies could not identify whether these observed links are causal. Evidence that probes the causality between BS and AD is urgently needed to establish preventive measures.

Mendelian randomization (MR) analysis, which is a method based on instrumental variables (IVs), has been widely used to assess the potential causality between exposure and outcome. Two recent MR studies suggested high and low BMI were not the causal risk factors for AD, but were both limited by survivor bias and BS (Mukherjee et al., 2015; Nordestgaard et al., 2017). Although Mukherjee et al. (2015) suggested that adiposity was not statistically associated with a risk of dementia and AD in sensitivity analyses, the effect of different BS on AD risk warrants further exploration. Therefore, we performed a large-scale MR analysis to assess the causal effect of BS on the risk of AD and simulation analyses to quantify the likely effect of BMI on risk of AD due to survivor bias.

## Materials and Methods

### Study Design

**FIGURE 1:**
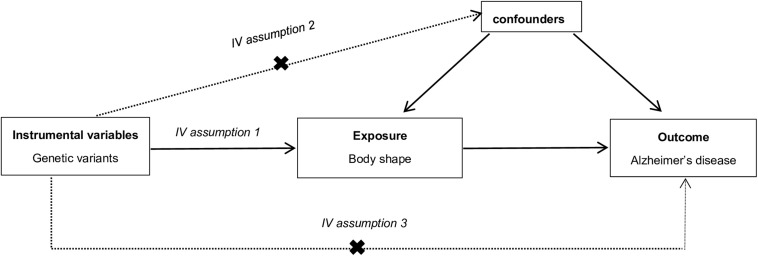
The explanation of Mendelian randomization analysis by a directed acyclic graph. The accuracy of estimating causality using Mendelian randomization (MR) analyses is based on the following three assumptions: (1) The instrumental variable (IV) associate robustly with the exposure (IV assumption 1). This assumption can be satisfied by ensuring *F* statistic > 10 and that SNPs are selected using genome-wide significance levels (*P* < 5 × 10^–8^), which suggests that potential bias from weak IV should not be substantial (Nordestgaard et al., 2017). (2) The IV is independent of combined influence of all confounders (IV assumption 2). For the same population and reference, we assess correlation of linkage disequilibrium between SNPs associated robustly with exposure and SNPs linked to possible known confounders. If the correlation coefficient is higher (i.e., *r*^2^ ≥ 0.5), the corresponding selected SNPs will be discarded (Geng et al., 2018). (3) The IV is independent of the outcome given the exposure and confounders (IV assumption 3). Horizontal pleiotropy, that IVs influence the outcome through alternative pathways other than the exposure, could violate this assumption. It can be checked by using MR-Egger regression and MR-PRESSO method (Noyce et al., 2017; Verbanck et al., 2018).

**FIGURE 2:**
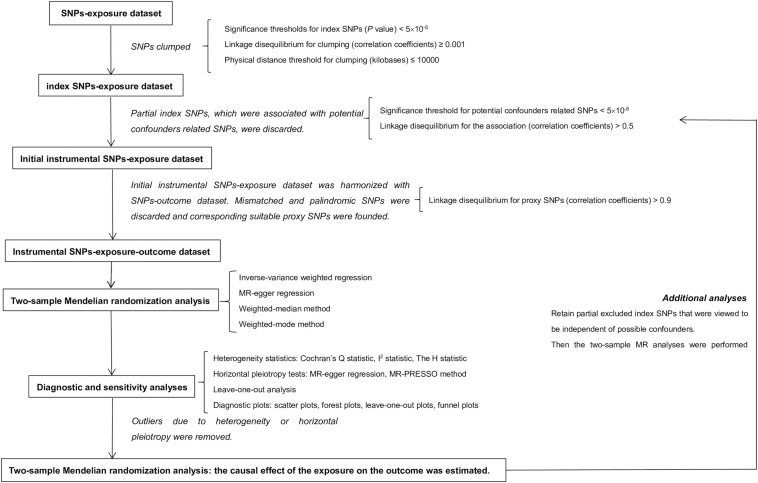
Study design of two-sample Mendelian randomization analysis. In this MR analysis, exposure and outcome refer to body shape and Alzheimer’s disease separately. MR, Mendelian randomization.

Compared with conventional observational analyses, MR analyses could provide stronger evidence regarding causal inference (Figure 1). MR analyses are analogous to randomized controlled trials (RCTs) and have been widely applied to investigate the potential causal relationship between exposure and the outcome variable. An individual’s genotype at a signal nucleotide polymorphism (SNP) was randomized at conception. This process is almost identical to an individual randomized to receive treatment or not in a RCT; therefore, an SNP could be considered to be an IV in MR analysis (Geng et al., 2018; Hemani et al., 2018). The study design of this MR analysis is mainly composed of the selection and validation of IVs, and the examination of causal effect between BS and risk of AD (Figure 2).

### Data Source

Two-sample MR analyses were conducted using genome-wide association study (GWAS) summary data. The two datasets were required to have a population of homogeneous characteristics (similar genetic ancestry), and in this study all participants were of European descent. This research did not require the consent of each participant because individual-level data were not used.

The association of SNPs with ATs were obtained from 2015 summary data from the Genetic Investigation of Anthropometric Traits (GIANT) consortium (Locke et al., 2015; Shungin et al., 2015). The dataset of associations of SNPs with BMI, WHR, WHRadjBMI, and WC includes a total of 2,554,637 SNPs in 322,154 individuals, 2,560,781 SNPs, 2,542,431 SNPs, and 2,565,407 SNPs in 210,088 individuals of European descent after imputation, respectively. The SNPs-AD dataset was from the stage I GWAS meta-analysis undertaken by the International Genomics of Alzheimer’s Project (IGAP), including genotype-AD associations for 7,055,881 SNPs after imputation in 17,008 AD cases and 37,154 controls of European descent (Lambert et al., 2013). However, sample overlap of the two datasets could bias the estimated causal effect. GWAS summary data contain meta-analyses of many larger and independent population-based cohorts; thus, the same cohort may be included in the SNPs-exposure and SNPs-outcome datasets simultaneously, i.e., sample overlap (Hemani et al., 2018). As a result, we estimated the degree of sample overlap between the SNPs-each AT GWAS dataset and the SNPs-AD study (Table 1). In addition, strong IVs (i.e., *F* statistic far greater than 10) could reduce bias from sample overlap.

**TABLE 1:** Possible sample overlap between the SNPs-each anthropometric trait and SNPs-Alzheimer’s disease datasets.

**Cohorts**	**Number of participants**				
	
	**AD**	**BMI**	**WHR**	**WHRadjBMI**	**WC**
AGES-RS	2772	3207	0	0	0
CHS	2255	3228	0	0	0
FHS	3344	8904	0	0	0
KORA F4	434	1811	0	0	0
Sum	8795	17150	0	0	0
Total European participants	54162	322,154	210,088	210,088	210,088
Proportion of participants of overlapping cohorts in total participants	16.24%	5.32%	0.00%	0.00%	0.00%

*AD, Alzheimer’s disease; BMI, body mass index; WC, waist circumference; WHR, waist-to-hip ratio; WHRadjBMI, waist-to-hip ratio adjusted for body mass index.*

Furthermore, the data in the two datasets for each SNP involved effect and other alleles, allele frequency of effect allele, beta-coefficient (SNPs-AD dataset: log-odds ratio [log-OR] of AD), standard error, and *P*-value, as well as corresponding chromosome and position.

### The Selection and Validation of Instrumental Variables

Instrumental Variables selected for exposure should be independent of each other, because linkage disequilibrium (LD) would introduce bias and lead to over-precise estimates in subsequent analysis, which could be ensured by clumping a large number of variants to a set of index SNPs (Noyce et al., 2017). Index SNPs were obtained by clumping all SNPs based on LD (*R*^2^ threshold of 0.001) or the physical distance threshold of 10,000 kb, while the robust associations between index SNPs and each AT reached genome-wide significance level (i.e., *P* < 5 × 10^–8^). After clumping, partial index SNPs were selected as IVs according to the following filtering conditions. First, the causal effects of BS on AD risk may be obscured by some known possible confounders; thus, the correlation coefficient (*r*^2^) between SNPs related to these confounders (i.e., Bonferroni-corrected *P* < 5 × 10^–8^) and IVs should be less than 0.5 (Lawlor et al., 2008). Second, it is widely acknowledged that three major alleles of apolipoprotein E (*APOE* ε2/ε3/ε4) are the most significant known genetic factors for AD in various ethnic groups, determined by rs429358 and rs7412 sites that should be irrelevant to IVs (Strittmatter et al., 1993). Index SNPs mismatching the above conditions were discarded and the remaining were selected as IVs. Furthermore, the statistical association between IVs and each AT was assessed using the *F* statistic, the calculation of which is detailed in the Supplementary Methods. The sub-dataset of IVs-each AT was then extracted and harmonized with the SNPs-AD dataset to obtain the corresponding IVs-each AT-AD dataset. However, some palindromic and ambiguous SNPs existed in the harmonized dataset and some SNPs selected from the AT dataset mismatched the AD dataset. Thus, these inappropriate SNPs were discarded and corresponding proxy SNPs were found. Suitable proxy SNPs must be in high LD with the inappropriate SNPs (*R*^2^ > 0.9), and irrelevant to SNPs that may have been associated with possible confounders and the *APOE* gene.

### Two-Sample Mendelian Randomization Analyses

For a single SNP, the Wald ratio is commonly used to derive the causal estimate (Thomas et al., 2007; Haycock et al., 2016). For multiple SNPs, inverse-variance weighted (IVW) linear regression, MR-egger regression, weighted-median method, and weighted-mode method were applied to obtain the overall causal estimate. Detailed descriptions of these methods are provided in the Supplementary Methods.

The two-sample analysis was performed as follows. First, the pooled causal estimates for multiple IVs were calculated only using the IVW and MR-egger methods (Burgess et al., 2013; Bowden et al., 2017). Diagnostic and sensitivity analyses were the implemented, after which there was no evidence of heterogeneity or horizontal pleiotropy. Second, four overall causal estimates for multiple IVs were separately derived. The MR analyses using MR-egger regression, weighted-median method, and weighted-mode method were regarded as sensitivity analyses to improve the reliability of the causal inference from the IVW method (Bowden et al., 2016; Hartwig et al., 2017). Third, the OR (95% CI) for risk of AD per 1-SD increase in each AT was reported because interpretation is easier using this metric than an arbitrary difference, while 1-SD increment separately represents a 4.69 kg/m^2^ increase in BMI, 0.14 cm/cm increase in WHR, 0.08 units increase in WHRadjBMI, and 12.06 cm increase in WC (Geng et al., 2018).

### Additional Analyses

These analyses were optional. When selecting IVs, partial index SNPs were removed because of the associations between them and potentially possible confounders. However, if these confounders were no longer regarded as confounders based on the results of the above MR analyses, the excluded SNPs were reconsidered as IVs. The MR analyses of BS and AD were conducted again to obtain more reliable results.

### Sensitivity Analyses

Diagnostic and sensitivity analyses were performed to identify different violations of assumptions and included tests of heterogeneity and horizontal pleiotropy, as well as four diagnostic plots. Heterogeneity statistics, including Cochran’s *Q*, *I*^2^, and *H* statistic, assessed heterogeneity in causal estimates from the IVW method (Higgins and Thompson, 2002; Higgins et al., 2003). A *P*-value of Cochran’s *Q* or *H* statistic less than 0.05 indicated heterogeneity in causal effects amongst all SNPs, while *I*^2^ values of 25, 50, and 75%, respectively, represent low, moderate, and high heterogeneity (Higgins et al., 2003). Test of horizontal pleiotropy was conducted using MR-egger regression and a Mendelian randomization pleiotropy residual sum and outlier (MR-PRESSO) approach. A non-zero intercept of the MR-egger regression suggested horizontal pleiotropy. In addition, the horizontal pleiotropic outliers that were identified using MR-PRESSO, along with outliers that were detected in a leave-one-out analysis, were removed (Verbanck et al., 2018). Moreover, phenome-wide association studies (Phewas) were used to interpret outliers and explore the potential reason why the IV assumptions were violated (Denny et al., 2010; Hemani et al., 2018). Four diagnostic plots, including a scatter plot, forest plot, leave-one-out plot, and funnel plot, were applied to present the findings of the MR analysis of BS and AD and aid in detecting horizontal pleiotropy and heterogeneity. Further detailed descriptions are given in the Supplementary Methods.

### Power Calculation

Statistical power is usually limited in MR analyses because of the lower variation in exposure explained by IVs and limited sample sizes, which could be computed using a published calculator (Brion et al., 2013; Jiang et al., 2018).

### Survivor Bias

Bias from selective mortality should be considered in epidemiological studies, especially in degenerative neurosis. In this MR analysis, BMI and age are widely acknowledged to be related to mortality, which may lead to a survivor bias and invalid causal estimate to some extent (Mayeda et al., 2016). Both low and high BMI are unfavorable to individual survival, and age is positively related to mortality; based on this, a simulation for quantifying the likely effect of survivor bias on the causal estimate was performed, assuming no association between BMI and AD. If the effect of BMI on AD risk due to a survivor bias reaches a significance level of 0.05, the real causal estimate is biased toward the null, i.e., BMI may be associated with AD. Otherwise, the real causal estimate is not influenced. Large sample sizes (*n* = 500,000) were generated in this simulation, along with data on BMI values, age, IVs, alive/dead status, and AD status for each individual (Davey et al., 2009; Qiu et al., 2009; Jodrá, 2009; Lambert et al., 2013).

This simulation was performed as follows (Noyce et al., 2017):

(1) Each individual was randomly assigned alleles at 60 instrumental SNPs based on corresponding real population-based allele frequencies. In other words, the genotype value of each SNP for each individual was generated and expressed as *g_*ij*_*, which represents the genotype value of SNP *j* (*j* = 1, 2, 3, …, 60) for individual *i* (*i* = 1, 2, 3, …, 500,000).
(2) Based on the created genotype values, the BMI value of each individual was generated as a function of allele frequencies of 60 instrumental SNPs and the beta coefficients of SNPs-BMI effects, plus a random number to obtain an individual difference, i.e., the BMI value of each individual could be simulated as follow: *x*_*i*_ = ∑*g*_*ij*_β_*j*_ + *e*_*j*_, where *e*_*j*_ followed a normal distribution with a mean of zero, and standard deviation of V_*E*_, where V_*E*_ was the residual phenotypic variance of BMI.
(3) We simulated the age for each individual through matching the age distribution reported in the AD meta-analysis (Lambert et al., 2013).
(4) The alive/dead status for each individual was modeled as a function of the age and BMI value of this individual. Age-related mortality as baseline mortality was generated from the Gompertz–Makeham law of mortality, based on parameters determined by previous studies. The baseline survival function was the obtained, with which the effect of BMI on survival was incorporated to acquire an age- and BMI-related survival function. According to this full survival model, the alive/dead status of individuals was simulated (Davey et al., 2009; Jodrá, 2009).

The cumulative distribution function of Gompertz–Makeham law of mortality is referred to as the Gompertz–Makeham distribution and can be described as follows:

$$F\,\left(t\right)=1-\exp\left(-\text{λ}\,t-\frac{a}{b}\,\left(e^{b\,t}-1\right)\right),$$

t>0,a,b,λ>0

The baseline survival function is derived through Gompertz–Makeham distribution and is as follows:

$$S_{b}\,\left(t\right)=1-F\,\left(t\right)=\exp\left(-\text{λ}\,t-\frac{a}{b}\,\left(e^{b\,t}-1\right)\right),$$

t>0,a,b,λ>0

The full survival model is*S*(*t*) = *S*_*b*_(*t*)*w*(*x*), where *w*(*x*) is a function of the impact of BMI on mortality.

(5) The AD status of individuals was generated according to the age-specific prevalence of AD obtained from a previously published study (Qiu et al., 2009). This simulation assumed that AD status was exclusively related to age and independent of BMI values.
(6) Two subsets were selected for further analysis. All individuals in the first subset were simulated without any death, thus all the analyses for this subset should be immune to the survivor bias. The second subset of AD cases and controls involved individuals who were confirmed as alive based on the full survival model. The observational studies and MR analyses were performed on the two subsets, respectively.
(7) The complete process was repeated 1000 times to produce two distributions of BMI-AD effects separately from the baseline and full survival models. The two distributions were compared to assess the degree to which the effect of BMI on AD could be driven by a survivor bias.

### Software and Packages

All analyses were conducted using R software (version 3.5.1). Based on a reference dataset of EUR population (1000 Genomes Project), the clumping procedure was implemented using R packages “Two-Sample MR” and “MRInstruments,” and the procedure of looking for proxy SNPs was conducted using R package “proxysnp.” The two-sample MR analysis was performed using R packages “Two-SampleMR,” “MRPRESSO,” and “Mendelian Randomization.”

## Results

### The Selection and Validation of Instrumental Variables

There were 69, 29, 38, and 42 index SNPs after clumping SNPs that, respectively, related to BMI, WHR, WHRadjBMI, and WC separately. Five of 69 index SNPs that were correlated with WHR-associated SNPs, while six of 29, none of 38, and 30 of 42 index SNPs that were correlated with BMI-associated SNPs were removed. None of the index SNPs correlated with the *APOE* gene. Additionally, some palindromic SNPs and mismatched SNPs were presented in the harmonized datasets, which were discarded and replaced by suitable proxy SNPs (Supplementary Table S1). Together 62 and 65 index SNPs for BMI, 23 and 29 index SNPs for WHR, and 12 and 41 index SNPs for WC remained in the first MR analysis and additional analysis, respectively, with 36 index SNPs for WHRadjBMI in the MR analysis. All *F* statistics showed far more than 10, which suggest it was unlikely for bias from weak instrument (Table 2). Therefore, these index SNPs were chosen as IVs for further MR analyses.

**TABLE 2:** The validation of instrumental variables.

**AT**	**The first analyses**			**Additional analyses**		
		
	**Remaining SNPs**	***R*^2^**	***F* statistic**	**Remaining SNPs**	***R*^2^**	***F* statistic**
BMI	62	1.66%	92.30	65	2.14%	114.17
WHR	23	0.59%	88.14	29	0.77%	91.71
WC	12	0.23%	65.54	41	1.42%	119.18
WHRadjBMI	36	1.13%	108.16			

*Remaining SNPs refer to the partial index SNPs after harmonizing the index SNPs-each AT dataset and the SNPs-Alzheimer’s disease dataset. AT, Anthropometric trait; BMI, body mass index; WHR, waist-to-hip ratio; WHRadjBMI, waist-to-hip ratio adjusted for body mass index; WC, waist circumference.*

### The First Mendelian Randomization Analyses

**FIGURE 3:**
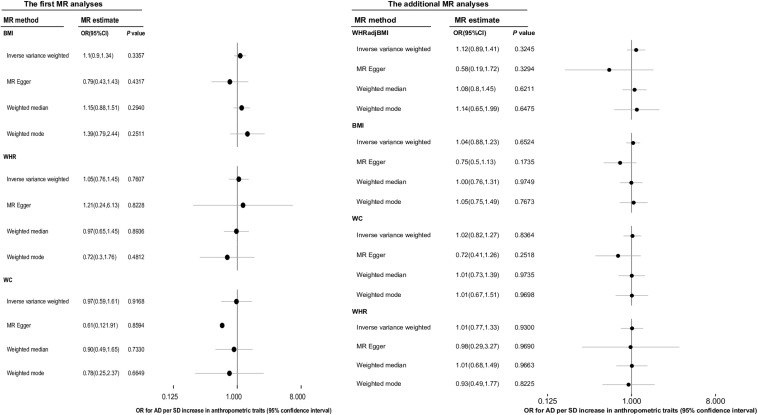
The overall causal effect of body shape on the risk of Alzheimer’s disease from each of four different methods (inverse-variance weighted method, MR-egger regression method, weighted-median method and weighted-mode method). Results were reported as the odd ratio (OR) of AD per 1-SD increase in each anthropometric trait. The results of additional MR analyses were the more reliable causal estimates of ATs on AD, and the causal effect of WHRadjBMI on AD was displayed in this column was just for easy reading. MR, Mendelian randomization; AD, Alzheimer’s disease; AT, anthropometric traits; BMI, body mass index; OR, odds ratio; WC, waist circumference; WHR, waist-to-hip ratio, WHRadjBMI, waist-to-hip ratio adjusted for body mass index.

In the MR analysis of BS and AD, the overall causal estimate from IVW method suggested no effect on risk of AD per SD change in each AT, which was the same as the results obtained using the other three methods comprising the MR-egger, weighted-mean, and weighted-mode methods (Figure 3). The OR (95% CI) of BMI, WHR, WHRadjBMI, and WC with respect to AD from IVW methods separately using 60, 23, 36, and 12 IVs was 1.10 (0.90, 1.34) (*P* = 0.336), 1.05 (0.76, 1.45) (*P* = 0.761), 1.12 (0.89, 1.41) (*P* = 0.324), and 0.97 (0.59, 1.61) (*P* = 0.917).

### Additional Mendelian Randomization Analyses

We conducted additional MR analyses as BMI and WHR were no longer considered confounders based on the results of the first MR analyses. The overall causal estimate [OR (95% CI)] for the effect of a 1-SD increase in BMI on the risk of AD using 65 IVs in the IVW method was 1.04 (0.88, 1.23) (*P* = 0.652), and for the effect of per 1-SD increase in WHR on the risk of AD using 29 IVs was 1.01 (0.77, 1.33) (*P* = 0.930). Moreover, there were 38 IVs remaining for WC after sensitivity analyses, which explained 1.33% of the variance in WC and the corresponding *F* statistic was 120.97. The OR (95% CI) of AD per 1-SD increase in WC calculated using the IVW method was 1.02 (0.82, 1.27) (*P* = 0.836). All the above results were in accordance with the conclusions of the other three methods (Figure 3).

### Power Calculation

We had sufficient statistical power to identify the moderate causal effect of BS on AD risk (Table 3).

**TABLE 3:** Statistical power in Mendelian randomization analyses of body shape and the risk of Alzheimer’s disease.

**Each AT**	***R*^2^**	**Statistical power**	**Minimal/Maximal detectable OR**
**BMI**			
Positive/negative correlated with AD risk	2.14%	81%	1.19/0.83
Previous association (Razay et al., 2006)	2.14%	>99%	1.80^∗^
**WHR**			
Positive/negative correlated with AD risk	0.77%	80%	1.33/0.73
Previous association (Razay et al., 2006)	0.77%	>99%	2.00^∗^
**WHRadjBMI**			
Positive/negative correlated with AD risk	1.13%	82%	1.27/0.77
**WC**			
Positive/negative correlated with AD risk	1.33%	80%	1.24/0.79

*Based on our sample size (case/control: 17,008/37,154, total: 54,162), minimal/maximal detectable odds ratio (OR) was calculated at a significance level of 0.05. *R*^2^ refers to the proportion of variance explained by IVs in each anthropometric trait. The statistical power of previous association in each AT means the power to detect previous association in this MR analysis. AD, Alzheimer’s disease; AT, anthropometric traits; BMI, body mass index; WC, waist circumference; WHR, waist-to-hip ratio; WHRadjBMI, waist-to-hip ratio adjusted for body mass index. ^∗^Association provided by a previous observational study.*

### Sensitivity Analyses

Finally, 65 IVs for BMI, 29 IVs for WHR, 36 IVs for WHRadjBMI and 38 IVs for WC were identified (Supplementary Table S2). For BMI, WHR, WHRadjBMI, and WC, the *I*^2^ value was 21.86, 20.46, 18.73, and 24.90%, respectively, while the *P*-value of the MR-PRESSO global test was 0.0674, 0.1688, 0.1577, and 0.0833, respectively. Sensitivity analyses of the first and additional MR analyses were implemented, and the estimates of causal effects were under the condition of no evidence of heterogeneity and horizontal pleiotropy (Supplementary Table S3 and Supplementary Figure S1).

### Simulation Analyses of Survivor Bias

**FIGURE 4:**
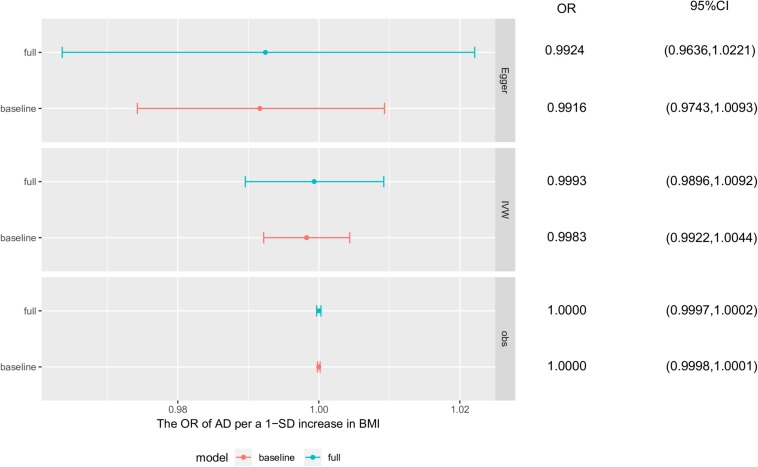
Survivor bias. The causal effects of these simulation analyses were obtained separately from base survivor model and full survivor model using observational study, inverse-variance weighted method and MR-egger method. Each point and horizontal line denote the mean estimated causal effect and 95% confidence interval (CI) from corresponding model and method. AD, Alzheimer’s disease; BMI, body mass index; baseline, baseline survivor model without survivor bias; full, full survivor model with selected mortality.

The simulation to assess the likely effect of survivor bias demonstrated no evidence that the causal estimate obtained using the IVW method was driven by survivor bias in the MR analysis of BMI and AD. Based on the results of observational studies and the MR analyses of the simulated dataset, the mean estimated causal effects and distribution of effects in the presence of survivor bias were similar to those without survivor bias, which both obeyed a Gaussian distribution. In conclusion, the result of the MR analysis using the IVW method compared with that using the MR-Egger method was more robust to bias from selected mortality (Figure 4 and Supplementary Figure S2).

## Discussion

Mendelian randomization analyses findings in this study suggested that BS has no influence on the risk of AD. To the best of our knowledge, there have been two MR studies of BMI and AD, which were limited by the possible influence from survivor bias and BS (Mukherjee et al., 2015; Nordestgaard et al., 2017). Mukherjee et al. (2015) reported the non-significance of the relationship between adiposity and AD by using mechanism-specific polygenic scores as IVs; however, their sensitivity analysis could not explore the influence of different BS on the risk of AD. This study of BMI and AD used large sample sizes to implement the MR analysis and simulation to explain the influence of survivor bias on the causal effect, with an MR analysis of WHRadjBMI being conducted, which demonstrated again that a causal effect of BMI on risk of AD, independent of survivor bias and BS, was not present. Additionally, to the best of our knowledge, this is the first MR analysis to explore the causal effect of BS on AD and no evidence of an effect was found.

Associations observed in available conventional epidemiologic evidence between BS and AD remained controversial (Supplementary Table S4). These observational studies defined an AT as a discrete or continuous variable and applied different methods to perform analyses, together with a study-specific bias, which contributed to the heterogeneity of the results. A follow-up study and cohort study indicated that low BMI was a risk factor of AD, whereas one case-control study found a U-shaped association (Razay et al., 2006; Hughes et al., 2009; Nordestgaard et al., 2017). Three cohort studies investigated the influence of BMI as a continuous variable on AD, two of which supported our results, and the other reported a positive correlation between them (Luchsinger et al., 2007; Luchsinger et al., 2012; Joo et al., 2018). Some studies proposed that lower BMI was linked with lower blood pressure, which mitigated the risk of AD, because hypertension could cause dysfunction of blood brain-barrier (BBB) via the formation of free oxygen radicals (Reitz et al., 2010; Nordestgaard et al., 2017). Other interpretations of the U-shaped association were that lower BMI was related to a higher risk of AD because of reverse causation or it was a predictor of preclinical AD, and associations of higher BMI with an elevated risk of AD could be considered as evidence that obesity was related to a greater risk of AD (Luchsinger et al., 2007, 2012). Moreover, two cohort studies showed a clear positive association between WHR as a continuous variable and risk of AD, contrary to the conclusions drawn by a case-control study (Razay et al., 2006; Reitz et al., 2010; Luchsinger et al., 2012). Two cohort studies concluded there was no association between WC and risk of AD (Luchsinger et al., 2007, 2012). Another observational study that performed multivariate analyses suggested that low BMI and WC were pre-clinical markers of AD (Chu et al., 2009).

We investigated the potential causal relationship between commonly used BS-related indexes, including BMI, WHR, WHRadjBMI, WC, and AD, and concluded that BS may play no causal role in AD. Regarding the relationship between obesity and AD, we further selected 18 obesity-related SNPs from 65 BMI-related SNPs as IVs to perform an MR analysis of obesity and AD, which explained 8.33% of the variance in obesity and the corresponding *F* statistic was 498.12. The ORs (95% CI) for AD per 1-SD increase in obesity calculated using the IVW method, MR-Egger method, weighted-median method, and weighted-mode method were, respectively, 0.97 (0.89, 1.04) (*P* = 0.387), 0.96 (0.77, 1.20) (*P* = 0.728), 1.00 (0.89, 1.12) (*P* = 0.974), and 1.02 (0.88, 1.17) (*P* = 0.824) separately. However, such findings together with the negative association of BMI and WHRadjBMI with AD (Figure 3) do not allow us to definitely conclude that obesity has no causal relationship with AD, which is often difficult to clarify. In fact, there often exist different pathways linking SNPs, obesity, and AD. Furthermore, it may be challenging to construct a reasonable counterfactual and causal inference model. Overall, exploring the real causal association and underlying mechanism between obesity and a disease such as AD is a challenging issue. Some explanations have been suggested and need to be examined in the future. Adiposity is closely related to stroke and vascular risk factors, such as hypertension, which could affect the deposition of amyloid β (Aβ), alter brain structure, and enhance BBB permeability. Meanwhile, many cytokines secreted by adipose tissue penetrate the BBB to influence normal brain function and thereby increase the risk of AD (Luchsinger et al., 2012; Nordestgaard et al., 2017). The association between AD and abdominal obesity was supported by a study that proposed higher WHR could affect brain normal structures and functionality through neurodegenerative, vascular, or metabolic processes (Jagust et al., 2005). However, this mechanism was thought to be unclear and partially explained by insulin resistance (Razay et al., 2006). Insulin resistance is always accompanied by type 2 diabetes and hyperinsulinemia. Diabetes may be related to AD through mediating oxidative stress and protein glycosylation (Mayeda et al., 2016); however, peripheral insulin could directly damage normal brain structures and functionality by crossing the BBB to act on insulin receptors located in the central nervous system (CNS). Insulin plays an important role in the up-regulation of extracellular Aβ levels and phosphorylation of tau protein, which could accelerate the process of AD (Mayeda et al., 2016). Another one study showed lower serum insulin-like growth factor-1 (IGF-1) levels were a risk factor for AD whereas higher IGF-1 levels could protect individuals against AD at the subclinical and clinical stages (Westwood et al., 2014). The evidence from conventional epidemiological studies regarding the effects of peripheral insulin and IGF-1 levels on the risk of AD has been weak and conflicting. Experimental findings showed that altered peripheral blood levels of insulin or IGF-1 were irrelevant to the normal functionality of the CNS, but insulin or IGF-1 locally produced in the CNS played a more important role in regulating CNS neuronal functions, corresponding with the consistent result of a recent two-sample MR analysis (Steen et al., 2005; Williams et al., 2018).

This study has several strengths. First, avoiding reverse causation, a large sample size, and sufficient statistical power were ensured in this two-summary data MR design to identify a causal reference. Second, various methods including IVW, MR-egger, weighted-median, and weighted-mode method were applied in the MR analyses, the causal estimates of which were consistent, thus increasing the robustness of our findings. Third, the influence of confounding factors on the causal estimate was weakened to some extent over the strict selection of IVs. For each AT and AD, the results from two MR analyses were consistent with each other, showing no evidence of a causal effect of BS on AD risk. The *APOE* gene that plays an important role in modulating the deposition of Aβ and neurofibrillary tangles of AD is the most common genetic cause of AD and was not in LD with any IVs (Ghayeghran et al., 2017). Fourth, the MR-egger regression and MR-PRESSO methods were both used to check the possible horizontal pleiotropy and identify outliers. Furthermore, the source of horizontal pleiotropy could be detected through the Phewas of outliers. Fifth, our MR analyses of BS and AD risk had sufficient statistical power to detect moderate causal effects as noted in observational studies. However, there are several limitations. First, the two-sample MR summary data analysis assumed a linear relationship between each AT and AD. Indeed, a U- or J-shaped association between each AT and related disease is common in observational studies (Cerhan et al., 2014). A non-linear causal effect warrants other methods that need individual level data (Burgess et al., 2014). Second, it was impossible to compare the difference in the causal estimates in subgroups using summary data. Third, Table 1 shows that there was a minimal proportion of sample overlap in this study, the influence of which could be minimized by using strong IVs. Finally, reasonable biological interpretations for the results of the MR analyses were not provided. Therefore, the results from these MR analyses could not be considered as a definite answer and should be generalized to the rest of the population with caution.

## Conclusion

In conclusion, the evidence from MR analyses showed no causal effect of BS on AD risk. An RCT is not feasible; hence, experimental findings from biological mechanisms are expected to provide a reasonable interpretation for these results. It is plausible and recommended that our findings are replicated in other MR studies with individual-level genotyping data and in other ethnic groups. Although BS is not associated with AD risk based on our results, maintaining body weight in the normal range is beneficial.

## Data Availability Statement

The datasets generated for this study can be found in the manuscript/Supplementary Materials. GIANT consortium data are available at https://portals.broadinstitute.org/collaboration/giant/index.php/GIANT_consortium. IGAP summary data are available at http://web.pasteur-lille.fr/en/recherche/u744/igap/igap_download.php and contains an application to be completed.

## Ethics Statement

Ethical review and approval was not required for the study on human participants in accordance with the local legislation and institutional requirements. Written informed consent for participation was not required for this study in accordance with the national legislation and the institutional requirements.

## Author Contributions

YZ performed the statistical analysis and wrote the first draft of the manuscript. XS and MZ wrote sections of the manuscript. All authors contributed to the conception and design of the study and manuscript revision, and read and approved the submitted version.

## Conflict of Interest

The authors declare that the research was conducted in the absence of any commercial or financial relationships that could be construed as a potential conflict of interest.

## Abbreviations

A β: Amyloid β
AD: Alzheimer’s disease
APOE: apolipoprotein E
AT: anthropometric trait
BBB: blood brain-barrier
BMI: body mass index
BS: body shape
CI: confidence interval
CNS: central nervous system
GIANT: Genetic Investigation of Anthropometric Traits
GWAS: genome-wide association study
IAGP: International Genomics of Alzheimer’s Project
IGF-1: insulin-like growth factor-1
IV: instrumental variable
IVW: inverse-variance weighted
LD: linkage disequilibrium
MR: Mendelian randomization
OR: odds ratio
Phewas: phenome-wide association studies
RCT: randomized controlled trial
SNP: signal nucleotide polymorphism
WC: waist circumference
WHR: waist-to-hip ratio
WHRadjBMI: waist-to-hip ratio adjusted for body mass index.
