# Supplementary material for: Body Shape and Alzheimer’s Disease: A Mendelian Randomization Analysis
Source: Front Neurosci. 2019 Oct 10;13:1084. doi: 10.3389/fnins.2019.01084 (PMC6795688; doi:10.3389/fnins.2019.01084)
Supplement: Supplementary file 1 [file Data_Sheet_1.PDF]

Supplementary Material

1 Supplementary Methods

The Calculation Method of F Statistic

F statistic could be calculated as follow: $F = \frac{R^2(n-1-k)}{(1-R^2)\times k}$, where R^2 referred to the proportion of explained variance by IVs in each AT, n the sample size and k the count of IVs. R^2 was calculated using the following formula: $R^2 = \sum 2\beta\left(1-f\right)f$, where *f* was the effect allele frequency of each SNP and beta (β) was the effect of each SNP on each AT, and R^2 was equal to sum the variance explained by each SNP in each AT (Locke et al., 2015).

Detailed Introductions of Five Mendelian Randomization Methods

Wald Ratio Method

Wald ratio method was generally applied to obtain the causal estimate for each single SNP, calculated by dividing the beta coefficient of the SNP-outcome effect by that of the SNP-exposure effect. And the SE of the Wald ratio was approximated by the delta method, which took into account the uncertainty separately in the SNP-exposure and the SNP-outcome association (Haycock et al., 2016; Thomas et al., 2007).

Inverse-variance Weighted Linear Regression

IVW linear regression was performed using conventional linear regression of the SNPs-exposure associations against SNPs-outcome associations, weighted by the inverse of the variance of the SNPs-outcome associations. The main assumption of this method is that each SNP is considered as a valid IV. The intercept from IVW linear regression is constrained to pass through the origin, and the slope is the overall causal estimate for the MR analysis with multiple IVs (Burgess et al., 2013; Hemani et al., 2018).

MR-egger Regression

MR-egger regression is the same as IVW linear regression except allowing a non-zero intercept. This method was used to evaluate the directional horizontal pleiotropy under an additional assumption that horizontal pleiotropy effects are independent of instrument strength (known as the INSIDE assumption). The intercept was compared with zero using a statistical hypothesis test at a significance level of 0.05. The intercept of MR-egger regression toward zero suggests no or balanced horizontal pleiotropy across all IVs, given the INSIDE assumption. A non-zero intercept indicates overall unbalanced horizontal pleiotropy and the violation of IV assumption 3, and under this circumstance, the slope from MR-egger regression is the corrected-pleiotropy causal estimate, while the causal

estimate from IVW method is biased. The advantage of the MR-egger method is that unbiased causal estimate could be provided assuming all selected IVs are invalid (Bowden et al., 2017).

Weighted-median Method

Unbiased causal estimate could be provided by the weighted-median method when at least 50% information came from valid IVs. Weights were given more to stronger IVs and were calculated through the inverse of the SE of the Wald ratio for each SNP (Bowden et al., 2016).

Weighted-mode Method

According to the similarity of causal estimates amongst all IVs, IVs were clustered into several groups, the largest of which was selected to derive a causal estimate. And if the IVs in this largest group are valid, the true causal estimate will be returned, and the SE is calculated through bootstrapping. Weights derive from the inverse of the variance of the SNPs-outcome associations. Compared with above three methods, the causal estimate from this method is robust to the influence of horizontal pleiotropy (Hartwig et al., 2017).

Diagnostic and Sensitivity Analyses

Heterogeneity Statistics

The statistical hypothesis tests for heterogeneity in causal effects amongst IVs were performed under the null assumption that the causal effect from each IV was identical. Heterogeneity statistics of meta-analysis were applied in the MR analyses involving Cochran's Q statistic, I^2 statistic and The H statistic.

(1) Cochran's Q statistic

Cochran's Q statistic was computed by summing the squared deviation of the causal estimate using each SNP from the overall causal estimate using multiple SNPs from IVW methods, with each SNP weighted by the inverse of the variance of the causal estimate. This statistic under the null hypothesis obeyed the chi-square distribution with *k*-1 degrees of freedom (where k referred to the number of SNPs). Cochran's Q statistic was computed using the following formula: $Q=\sum_{i=1}^{k} w_i (T_i - \overline{T})^2$, $\overline{T}=\frac{\sum w_i T_i}{\sum w_i}$, where w_i referred to the weight of the contribution of SNP *i* (*i* = 1,2,3…*k*) to the overall estimate, T_i the causal estimate obtained using SNP *i*, $\overline{T}$ the overall causal estimate obtained using SNPs of *k* numbers from IVW method (Higgins et al., 2003).

(2) I^2 statistic

I^2 statistic could be helpful in quantify the effect of heterogeneity, which describes the total variation due to heterogeneity across causal estimates amongst all SNPs. Based on Cochran's Q statistic, I^2 could be calculated as follow: $I^2 = 100\% \times (Q - df)/Q$. Larger I^2 statistic indicates the greater heterogeneity, and it is generally recognized that I^2 values of 25%, 50% and 75% respectively represents low, moderate and high heterogeneity (Higgins et al., 2003).

(3) The H statistic

Based on Cochran's Q statistic, the H statistic could be calculated as follow: $H = \sqrt{\frac{Q}{k-1}}$. And the 95% CI is of the form: $\exp(\text{In}(H) \pm Z_{\alpha} \times SE[\text{In}(H)])$, where Z_{α} is the (1−α/2) quantiles of the standard normal distribution and $SE[\text{In}(H)] = \frac{\text{In}(Q) - \text{In}(k-1)}{2\left[\sqrt{2Q} - \sqrt{(2k-3)}\right]}$ when Q > k, $SE[\text{In}(H)] = \sqrt{\left\{\frac{1}{2(k-2)}\left[1 - \frac{1}{3(k-2)^2}\right]\right\}}$ when Q ≤ k. H = 1 indicates no heterogeneity in causal effects amongst all SNPs, and generally, H < 1.2 could be accepted. H > 1.5 suggests evidence of heterogeneity across causal estimates. That H = 1 is involved in 95% CI of the H statistic implies no statistical heterogeneity (Higgins and Thompson, 2002).

Horizontal Pleiotropy Tests

Horizontal pleiotropy could be evaluated by MR-egger regression and MR-PRESSO method. The intercept of MR-egger regression was compared with zero to identify whether unbalance horizontal pleiotropy emerges, about which details has been discussed above. The MR-PRESSO approach was developed to detect and correct horizontal pleiotropic outliers, which included the following three parts: (a) the MR-PRESSO global test evaluated overall horizontal pleiotropy under the null hypothesis of no horizontal pleiotropy; (b) the MR-PRESSO outlier test was performed to identify horizontal pleiotropic outliers. (c) the MR-PRESSO distortion test was performed to examine whether the causal estimate from IVW method was biased by identified outliers (Verbanck et al., 2018).

Leave-one-out Analyses

Leave-one-out analyses were performed to evaluate whether the overall causal estimates from IVW method were biased or driven by an outlier that may due to horizontal pleiotropy or heterogeneity. We conducted this analysis of each AT and AD through re-calculating the overall causal estimate by sequentially dropping one SNP at a time, and if the outlier was dropped, the new causal estimate would be statistically different with the original causal estimate.

Diagnostic Plots

There were four different diagnostic plots to depict the robustness of the causal effect in the MR analysis, including a scatter plot, forest plot, leave-one-out plot and funnel plot (Hemani et al., 2018). The brief descriptions are provided below. The scatter plot visualizes the relationship of SNPs-each AT associations against SNPs-AD associations, in which the slope of regression refers to the overall causal estimate of each AT on the risk of AD using multiple instrumental SNPs. The forest plot is used to present the contribution of each SNP to the overall causal estimate. The leave-one-out plot is similar to the forest plot, which is performed to visualize the result of the leave-one-out analysis. Both the forest and the leave-one-out plot are of help to detect the outliers due to heterogeneity. The funnel plot depicts instrumental strength against the causal estimate using each SNP, in which asymmetry suggests the invalid IVs and the violation of IV assumption.

REFERENCES

Bowden, J., Davey, S. G., Haycock, P. C., Burgess, S. (2016). Consistent Estimation in Mendelian Randomization with Some Invalid Instruments Using a Weighted Median Estimator. *GENET EPIDEMIOL*. 40, 304-314. doi: 10.1002/gepi.21965

Bowden, J., Del Greco M, F., Minelli, C., Davey Smith, G., Sheehan, N., Thompson, J. (2017). A framework for the investigation of pleiotropy in two-sample summary data Mendelian randomization. *STAT MED*. 36, 1783-1802. doi: 10.1002/sim.7221

Burgess, S., Butterworth, A., Thompson, S. G. (2013). Mendelian Randomization Analysis With Multiple Genetic Variants Using Summarized Data. *GENET EPIDEMIOL*. 37, 658-665. doi: 10.1002/gepi.21758

Hartwig, F. P., Davey Smith, G., Bowden, J. (2017). Robust inference in summary data Mendelian randomization via the zero modal pleiotropy assumption. *INT J EPIDEMIOL*. 46, 1985-1998. doi: 10.1093/ije/dyx102

Haycock, P. C., Burgess, S., Wade, K. H., Bowden, J., Relton, C., Davey Smith, G. (2016). Best (but oft-forgotten) practices: the design, analysis, and interpretation of Mendelian randomization studies. *The American Journal of Clinical Nutrition*. 103, 965-978. doi: 10.3945/ajcn.115.118216

Hemani, G., Zheng, J., Elsworth, B., Wade, K. H., Haberland, V., Baird, D., et al. (2018). The MR-Base platform supports systematic causal inference across the human phenome. *ELIFE*. 7. doi: 10.7554/eLife.34408

Higgins, J. P. T., Thompson, S. G. (2002). Quantifying heterogeneity in a meta-analysis. *STAT MED*. 21, 1539-1558. doi: 10.1002/sim.1186

Higgins, J. P., Thompson, S. G., Deeks, J. J., Altman, D. G. (2003). Measuring inconsistency in meta-analyses. *BMJ*. 327, 557-560. doi: 10.1136/bmj.327.7414.557

Locke, A. E., Kahali, B., Berndt, S. I., Justice, A. E., Pers, T. H., Day, F. R., et al. (2015). Genetic studies of body mass index yield new insights for obesity biology. *NATURE*. 518, 197-206. doi: 10.1038/nature14177

Thomas, D. C., Lawlor, D. A., Thompson, J. R. (2007). Re: Estimation of Bias in Nongenetic Observational Studies Using "Mendelian Triangulation" by Bautista et al. *ANN EPIDEMIOL*. 17, 511-513. doi: 10.1016/j.annepidem.2006.12.005

Verbanck, M., Chen, C., Neale, B., Do, R. (2018). Detection of widespread horizontal pleiotropy in causal relationships inferred from Mendelian randomization between complex traits and diseases. *NAT GENET*. 50, 693-698. doi: 10.1038/s41588-018-0099-7

2 Supplementary Figures and Tables

2.1 Supplementary Figures

Supplementary Figure 1. Diagnostic plots for the Mendelian randomization analysis of the causal effect of body shape on the risk of Alzheimer's disease. (A-G)

Figure A-G presented the diagnostic plots for the Mendelian randomization (MR) analysis of the causal effect of an anthropometric trait (BMI/WHR/WHRadjBMI/WC) on the risk of Alzheimer's disease (AD). Figure A-D visualized the diagnostic plots of the first MR analyses, while Figure E-G visualized these of the second MR analyses that are additional analyses. (a) A scatter plot. Each black point represents the relationship between the association of an instrumental SNP and this anthropometric trait against the association of it and AD risk, and corresponding error bar represents 95% confidence intervals (CI). The slope of each regression line represents the causal effect from corresponding MR method. (b) A forest plot. Each black point and horizontal line separately denote the causal effect obtained by Wald ratio method and 95% CI. The red points and horizontal lines in the bottom separately denote the overall causal effect obtained by each of two different methods (inverse-variance weighted (IVW) method and MR-egger method) and 95% CI. (c) A Funnel plot. Each black point represents the relationship between the causal effect from Wald ratio method against the inverse of the standard error of it, and the vertical lines represent the overall causal effect from each of two different methods (IVW method and MR-egger method). (d) A leave-one-out plot. Each black point denotes the causal effect by IVW method after removing the specific SNP on the left side, and the red point denotes the overall causal effect from IVW method using all SNPs. Horizontal lines denote 95% CI.

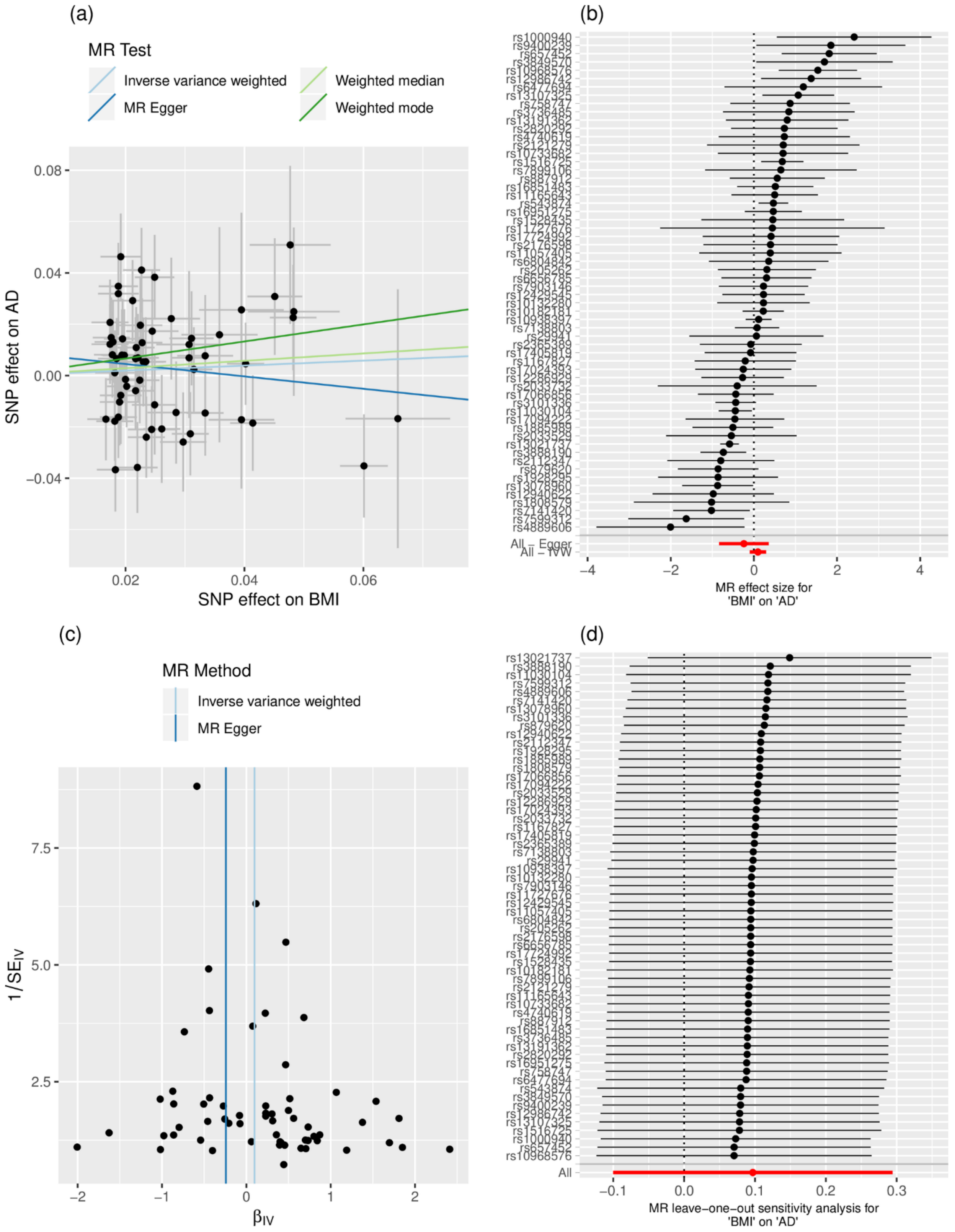

Figure A. Diagnostic plots for a Mendelian randomization analysis of the causal effect of body mass index on the risk of Alzheimer’s disease. There are totally 60 instrumental SNPs for BMI.

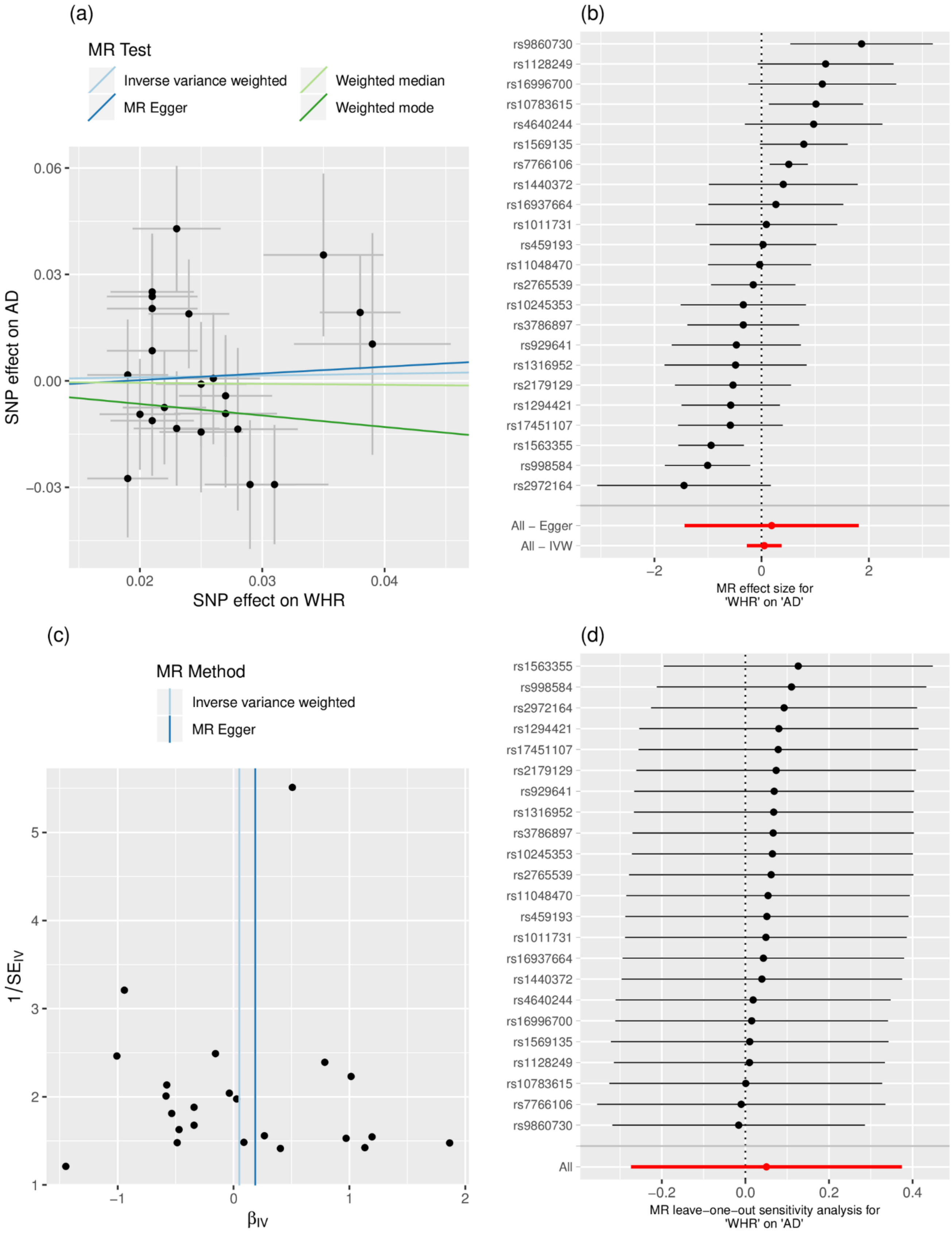

Figure B. Diagnostic plots for a Mendelian randomization analysis of the causal effect of waist-to-hip ratio on the risk of Alzheimer's disease. There are totally 23 instrumental SNPs for WHR.

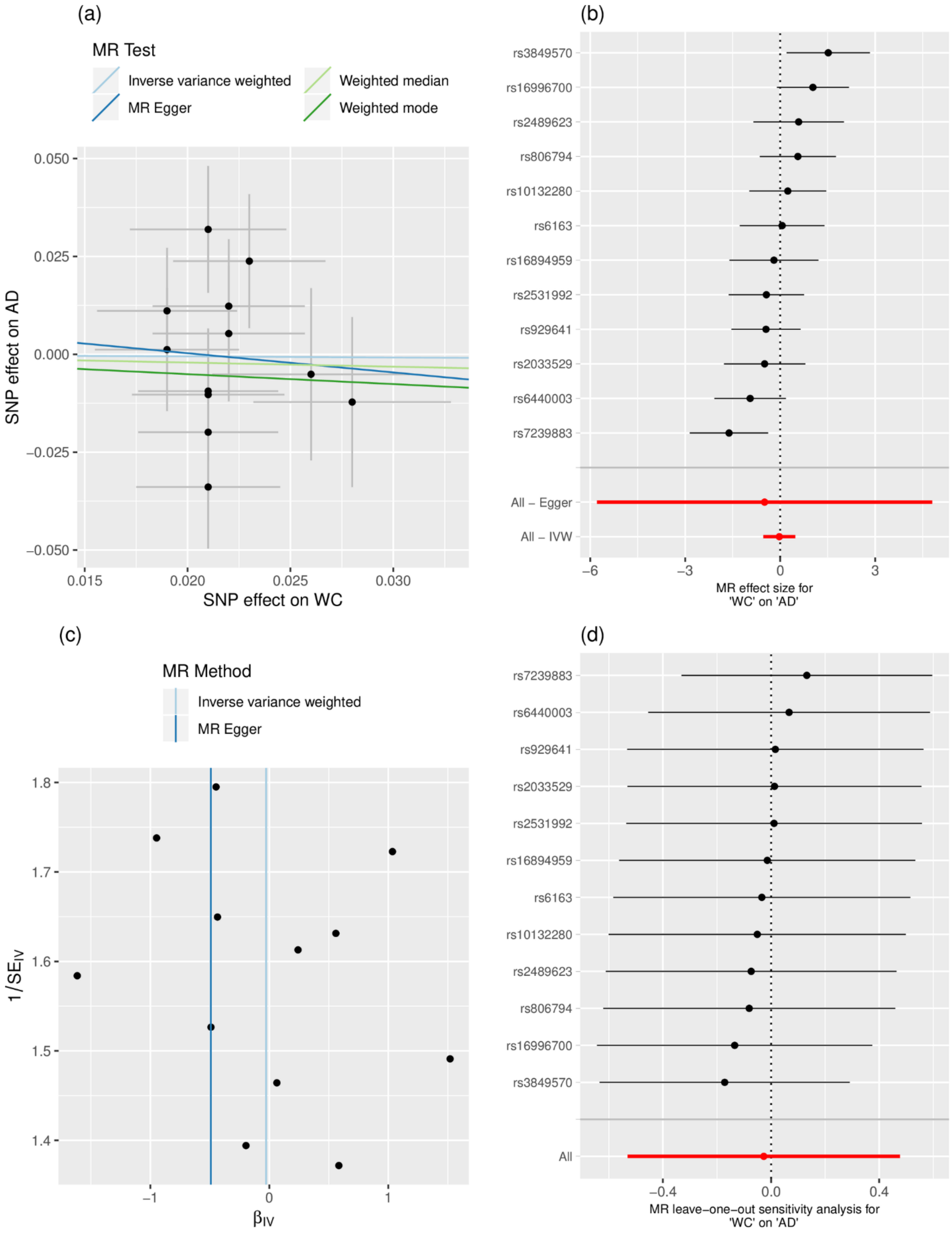

Figure C. Diagnostic plots for a Mendelian randomization analysis of the causal effect of waist circumference on the risk of Alzheimer's disease. There are totally 12 instrumental SNPs for WC.

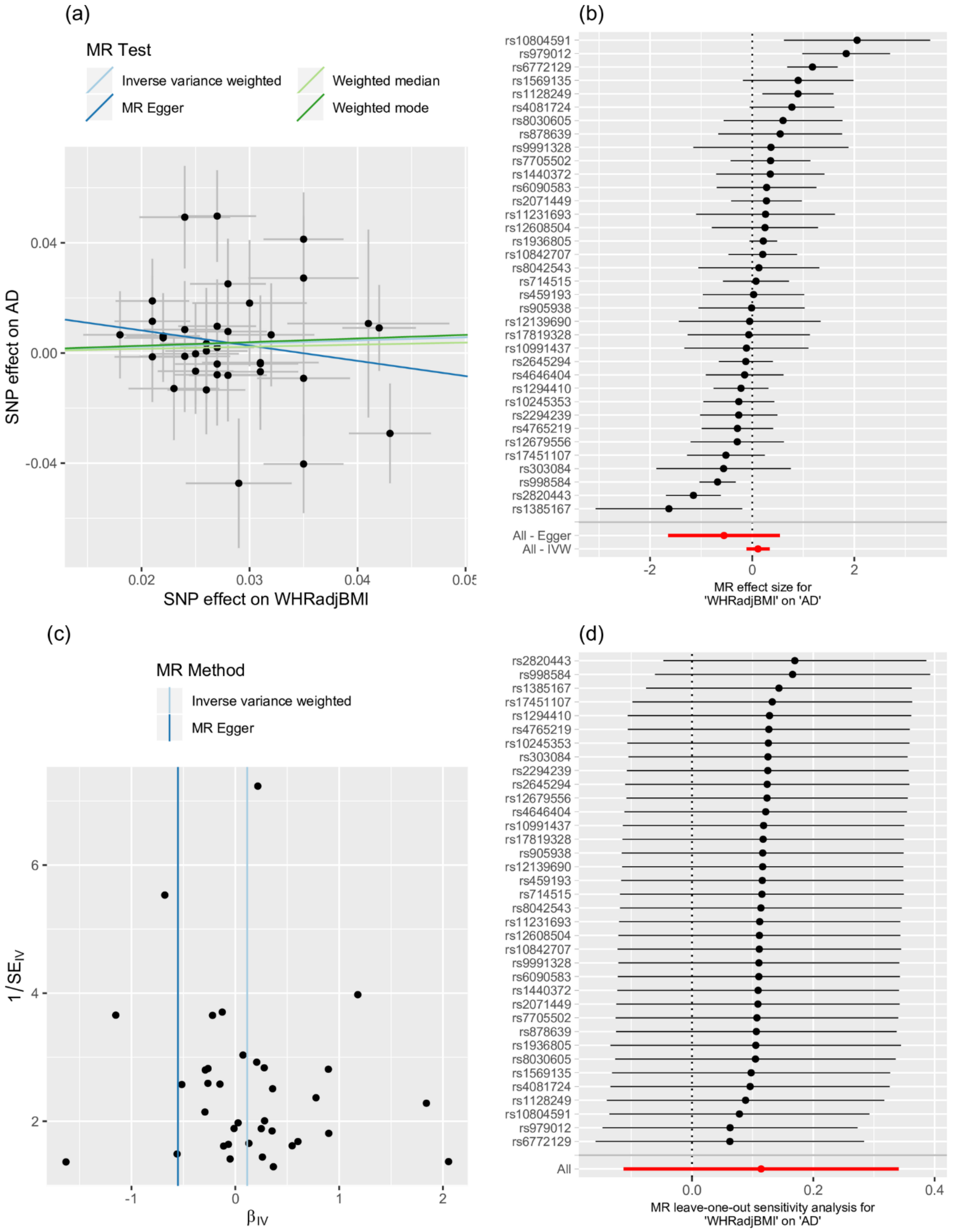

Figure D. Diagnostic plots for a Mendelian randomization analysis of the causal effect of waist-to-hip adjusted for body mass index on the risk of Alzheimer's disease. There are totally 36 instrumental SNPs for WHRadjBMI.

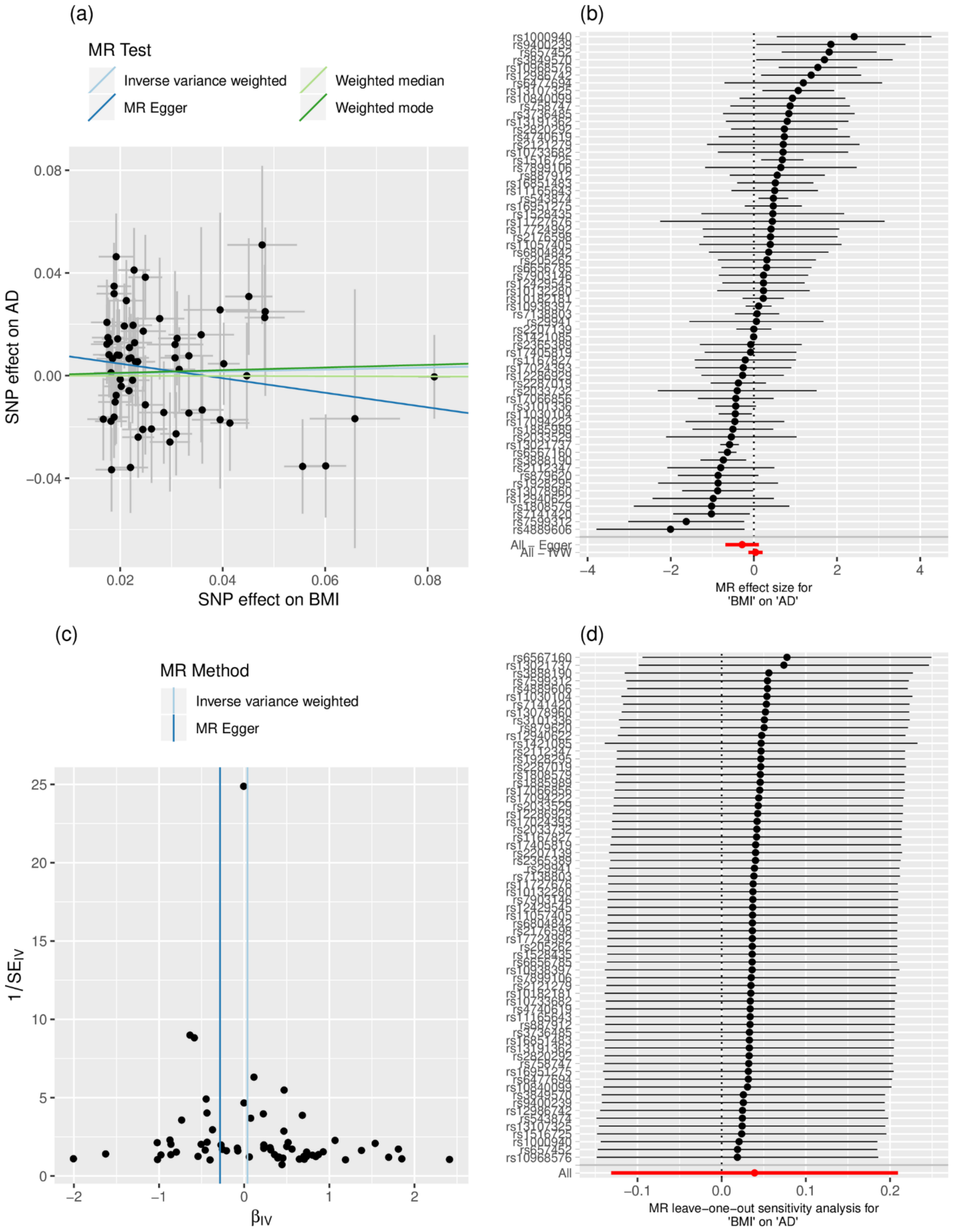

Figure E. Diagnostic plots for a Mendelian randomization analysis of the causal effect of body mass index on the risk of Alzheimer's disease. There are totally 65 instrumental SNPs for BMI.

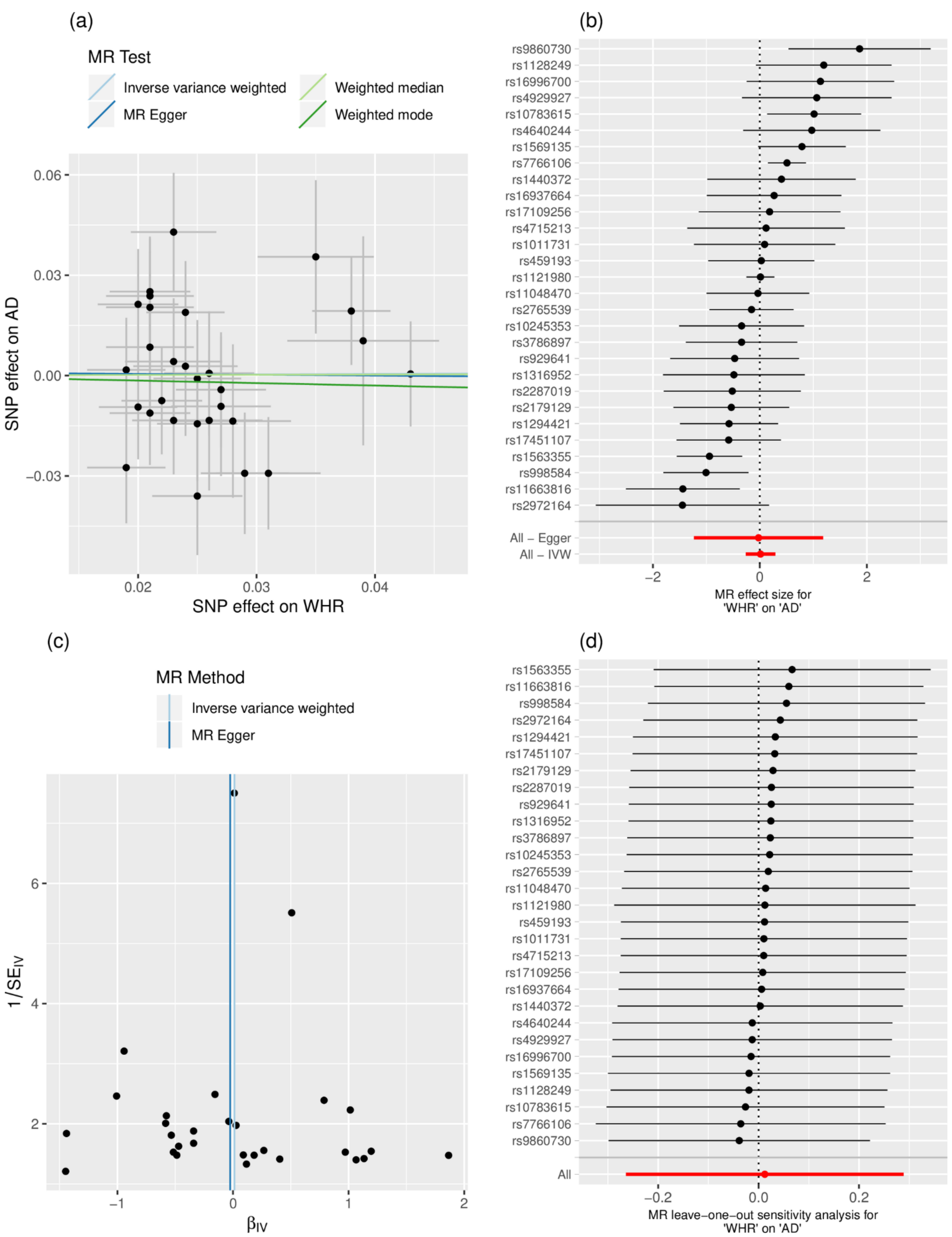

Figure F. Diagnostic plots for a Mendelian randomization analysis of the causal effect of waist-to-hip ratio on the risk of Alzheimer's disease. There are totally 29 instrumental SNPs for WHR.

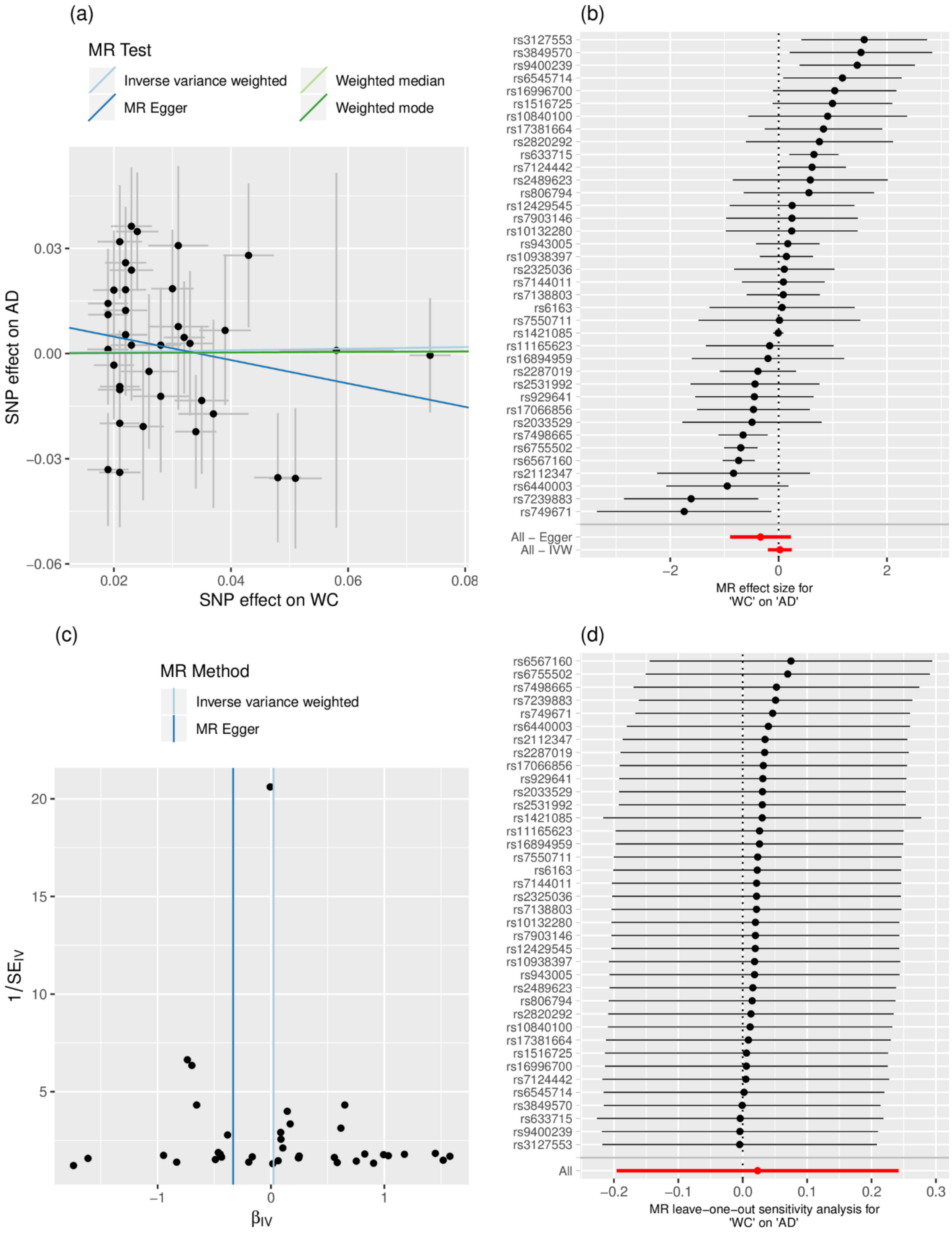

Figure G. Diagnostic plots for a Mendelian randomization analysis of the causal effect of waist circumference on the risk of Alzheimer's disease. There are totally 38 instrumental SNPs for WC.

Supplementary Figure 2. Distribution of the causal effects of BMI on AD risk separately obtained from baseline survivor model and full survivor model using three methods (observational study, inverse-variance weighted method and MR-egger method). (A-C)

Figure A-C presents the distribution of the causal effects of BMI on AD risk separately obtained from baseline survivor model and full survivor model using three methods. This distribution was based on 1000 repeated simulations. This figure could be used to compare the robustness of three methods (observational studies, inverse-variance weighted (IVW) method and MR-egger method) to survivor bias. AD, Alzheimer’s disease; baseline, baseline survivor model without survivor bias; full, full survivor model with selected mortality; MR, Mendelian randomization.

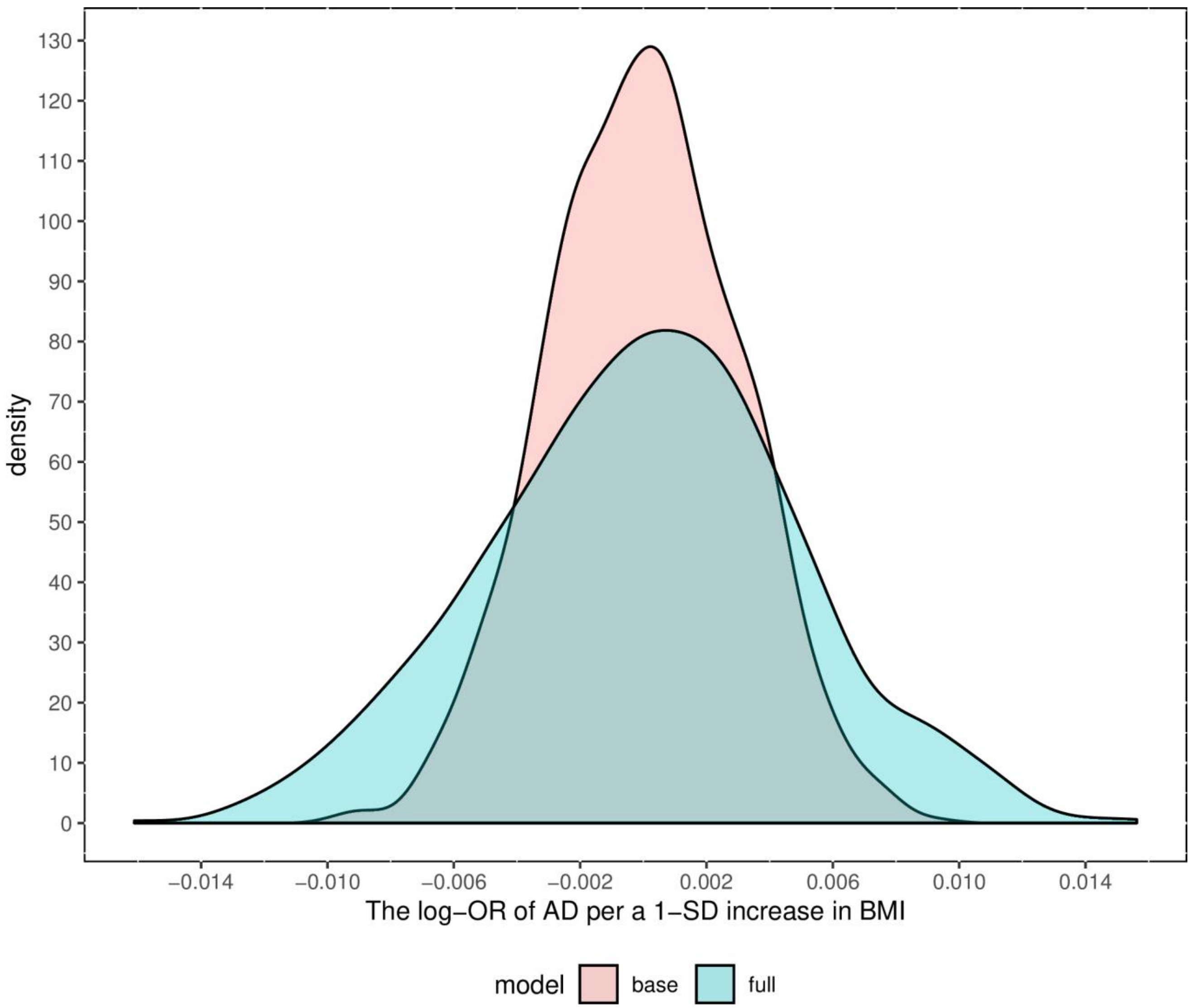

Figure A. Observational studies of baseline survival model and full survival model.

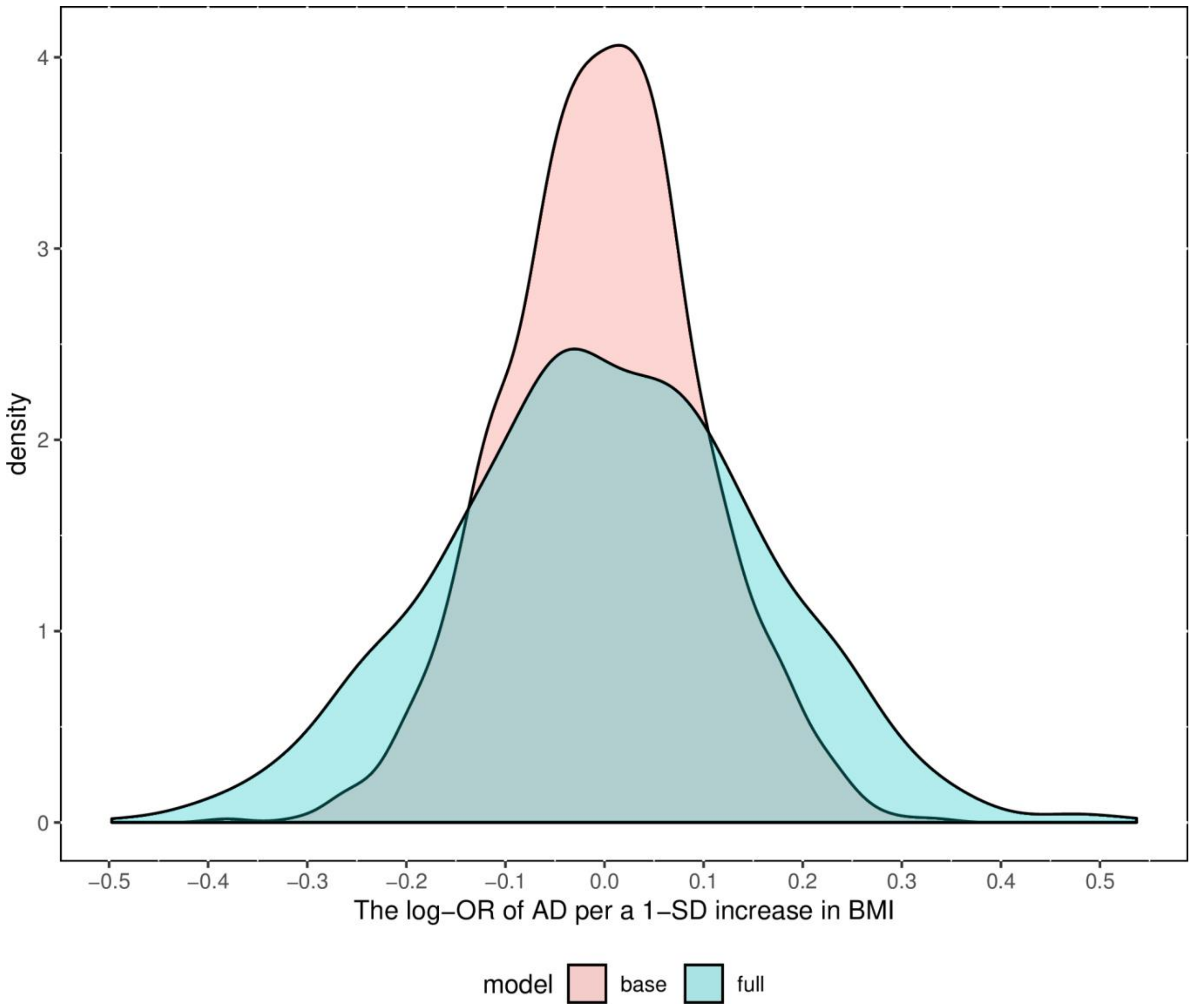

Figure B. MR analysis of baseline survival model and full survival model using IVW method.

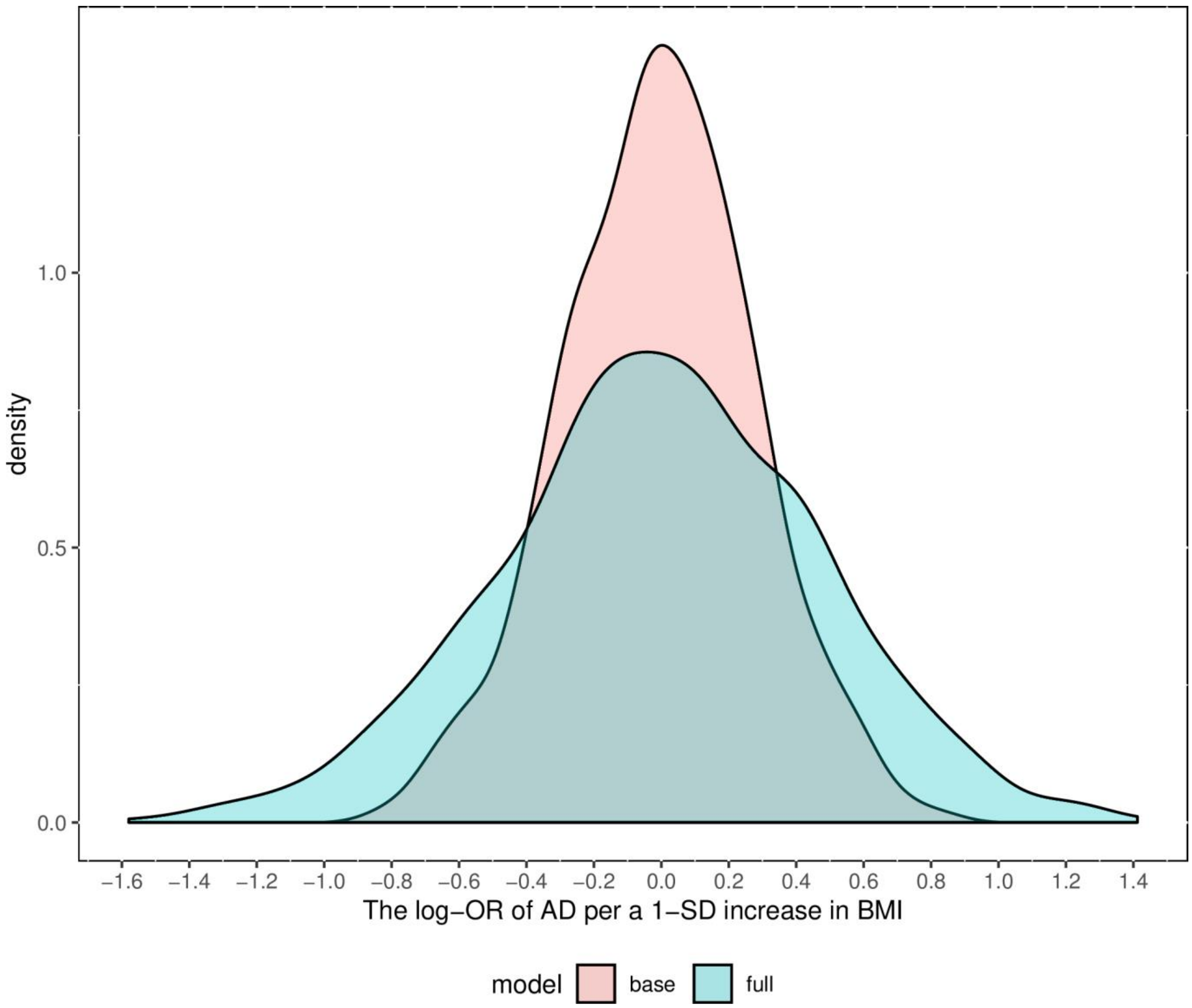

Figure C. MR analysis of baseline survival model and full survival model using MR-egger method.

2.2 Supplementary Tables

Supplementary Table 1. Partial index SNPs discarded and corresponding proxy SNPs found in the process of harmonizing the index SNPs-each AT dataset and the SNPs-AD dataset.

Anthropometric traits	Index SNP	Index SNP_EA/OA	Index SNP_MAF	Cause	Proxy SNP	Proxy SNP_EA/OA	Proxy SNP_MAF	R.squared	D.prime
BMI	rs17001654	C/G	0.8417	palindromic	-	-	-	-	-
BMI	rs9579083	G/C	0.7667	palindromic	rs1885989	G/A	0.1938	0.956	0.987
BMI	rs1016287	T/C	0.325	mismatched	rs887912	T/C	0.2803	0.990	0.995
BMI	rs2245368	T/C	0.7583	mismatched	-	-	-	-	-
BMI	rs1558902	A/T	0.45	palindromic	rs1421085	C/T	0.45	1	1
BMI	rs4256980	G/C	0.725	palindromic	rs10840099	C/T	0.725	0.996	1
WHR	rs9491696	G/C	0.525	palindromic	rs7766106	C/T	0.4573	0.996	1
WHR	rs12549058	G/T	0.0583	mismatched	rs16937664	C/T	0.0855	1	1
WHRadjBMI	rs12143789	C/G	0.1583	palindromic	rs12139690	A/G	0.1660	0.993	1
WHRadjBMI	rs2276824	C/G	0.4833	palindromic	-	-	-	-	-
WHRadjBMI	rs7759742	A/T	0.525	mismatched	-	-	-	-	-
WC	rs10767658	C/G	0.3583	palindromic	rs7124442	C/T	0.3667	0.962	1
WC	rs1558902	A/T	0.45	palindromic	rs1421085	C/T	0.45	1	1
WC	rs4776970	T/A	0.3417	palindromic	-	-	-	-	-

Cause indicates the reason why this specific index SNP was discarded. Correlation coefficient (R.squared) and D.prime both are used to quantify the level of linkage disequilibrium between index SNPs and proxy SNPs. AD, Alzheimer's disease; AT, anthropometric trait; BMI, body mass index; EA/OA, effect allele/other allele; MAF, effect allele frequency; WC, waist circumference; WHR, waist-to-hip ratio; WHRadjBMI, waist-to-hip ratio adjusted for body mass index. "-", no suitable proxy SNP was found.

Supplementary Table 2. Instrumental SNPs for each anthropometric trait and the association between these and AD risk. (A-D)

Table A. Instrumental SNPs for BMI and the association between these and AD risk.

SNP	Chr	BP	EA	OA	EAF	beta_BMI	SE_BMI	*P* value_BMI	log-OR_AD	SE_log-OR AD	*P* value_AD
rs1000940	17	5283252	G	A	0.225	0.0192	0.0034	1.28×10^{-08}	0.0463	0.0168	0.005767
rs10132280	14	25928179	A	C	0.3333	-0.023	0.0034	1.14×10^{-11}	-0.0053	0.0173	0.7571
rs10182181	2	25150296	A	G	0.5	-0.0307	0.0031	8.78×10^{-24}	-0.0069	0.0154	0.6548
rs10733682	9	129460914	A	G	0.425	0.0174	0.0031	1.83×10^{-08}	0.0122	0.0154	0.4287
rs10938397	4	45182527	A	G	0.5667	-0.0402	0.0031	3.21×10^{-38}	-0.0046	0.016	0.774
rs10968576	9	28414339	G	A	0.2917	0.0249	0.0033	6.61×10^{-14}	0.0383	0.0165	0.02045
rs11030104	11	27684517	A	G	0.8	0.0414	0.0038	5.56×10^{-28}	-0.0185	0.0186	0.3202
rs11057405	12	122781897	A	G	0.0917	-0.0307	0.0055	2.02×10^{-08}	-0.0121	0.0286	0.6712
rs11165643	1	96924097	C	T	0.425	-0.0218	0.0031	2.07×10^{-12}	-0.0109	0.0158	0.4886
rs1167827	7	75163169	A	G	0.4583	-0.0202	0.0033	6.33×10^{-10}	0.0042	0.0159	0.7909
rs11727676	4	145659064	C	T	0.075	-0.0358	0.0064	2.55×10^{-08}	-0.0159	0.0419	0.7044
rs12286929	11	115022404	G	A	0.4333	0.0217	0.0031	1.31×10^{-12}	-0.0059	0.0154	0.7008
rs12429545	13	54102206	G	A	0.9	-0.0334	0.0047	1.09×10^{-12}	-0.0077	0.0237	0.7451
rs12940622	17	78615571	A	G	0.4583	-0.0182	0.0031	2.49×10^{-09}	0.0178	0.0154	0.2487
rs12986742	2	58975143	C	T	0.5	0.0212	0.0037	1.01×10^{-08}	0.0292	0.0158	0.06422
rs13021737	2	632348	A	G	0.125	-0.0601	0.004	1.11×10^{-50}	0.0352	0.0201	0.07906
rs13078960	3	85807590	T	G	0.8167	-0.0297	0.0039	1.74×10^{-14}	0.0259	0.0193	0.1792
rs13107325	4	103188709	C	T	0.8833	-0.0477	0.0068	1.83×10^{-12}	-0.0509	0.0308	0.09826
rs13191362	6	163033350	A	G	0.8	0.0277	0.0048	7.34×10^{-09}	0.0222	0.0237	0.3473
rs1516725	3	185824004	T	C	0.0917	-0.0451	0.0046	1.89×10^{-22}	-0.0308	0.0227	0.1761
rs1528435	2	181550962	T	C	0.5833	0.0178	0.0031	1.20×10^{-08}	0.0081	0.0166	0.6258

SNP	Chr	BP	EA	OA	EAF	beta_BMI	SE_BMI	*P* value_BMI	log-OR_AD	SE_log-OR AD	*P* value_AD
rs16851483	3	141275436	G	T	0.9083	-0.0483	0.0077	3.55×10^{-10}	-0.0249	0.0328	0.4489
rs16951275	15	68077168	C	T	0.225	-0.0311	0.0037	1.91×10^{-17}	-0.0145	0.0183	0.4283
rs17024393	1	110154688	C	T	0.04167	0.0658	0.0088	7.03×10^{-14}	-0.0168	0.0504	0.7384
rs17066856	18	58049656	C	T	0.1333	-0.0395	0.0055	6.23×10^{-13}	0.0172	0.0268	0.5202
rs17094222	10	102395440	C	T	0.2083	0.0249	0.0038	5.94×10^{-11}	-0.0114	0.0193	0.5564
rs17405819	8	76806584	C	T	0.3667	-0.0224	0.0033	2.07×10^{-11}	0.0018	0.0168	0.9162
rs17724992	19	18454825	A	G	0.6917	0.0194	0.0035	3.42×10^{-08}	0.008	0.0177	0.6504
rs1808579	18	21104888	T	C	0.475	-0.0167	0.0031	4.17×10^{-08}	0.017	0.016	0.2876
rs1928295	9	120378483	C	T	0.425	-0.0188	0.0031	7.91×10^{-10}	0.0162	0.0159	0.3077
rs2033529	6	40348653	G	A	0.2583	0.019	0.0033	1.39×10^{-08}	-0.0103	0.0169	0.5434
rs2033732	8	85079709	C	T	0.7583	0.0192	0.0035	4.89×10^{-08}	-0.0077	0.0189	0.6833
rs205262	6	34563164	A	G	0.7333	-0.0221	0.0035	1.75×10^{-10}	-0.0069	0.0171	0.6883
rs2112347	5	75015242	G	T	0.375	-0.0261	0.0031	6.19×10^{-17}	0.0208	0.021	0.3241
rs2121279	2	143043285	T	C	0.1167	0.0245	0.0044	2.31×10^{-08}	0.0173	0.0235	0.461
rs2176598	11	43864278	T	C	0.2	0.0198	0.0036	2.97×10^{-08}	0.0079	0.0179	0.6599
rs2365389	3	61236462	C	T	0.6583	0.02	0.0031	1.63×10^{-10}	-0.0015	0.0158	0.9224
rs2820292	1	201784287	A	C	0.4917	-0.0195	0.0031	1.83×10^{-10}	-0.0143	0.0156	0.3611
rs29941	19	34309532	A	G	0.3333	-0.0182	0.0033	2.41×10^{-08}	-0.0011	0.0165	0.9468
rs3101336	1	72751185	T	C	0.3509	-0.0334	0.0031	2.66×10^{-26}	0.0146	0.0166	0.3778
rs3736485	15	51748610	A	G	0.425	0.0176	0.0031	7.41×10^{-09}	0.0148	0.0156	0.3421
rs3849570	3	81792112	A	C	0.3667	0.0188	0.0034	2.60×10^{-08}	0.0319	0.0162	0.04886
rs3888190	16	28889486	A	C	0.3583	0.0309	0.0031	3.14×10^{-23}	-0.0227	0.0162	0.1615
rs4740619	9	15634326	T	C	0.5333	0.0179	0.0031	4.56×10^{-09}	0.0131	0.0159	0.4082

SNP	Chr	BP	EA	OA	EAF	beta_BMI	SE_BMI	*P* value_BMI	log-OR_AD	SE_log-OR AD	*P* value_AD
rs4889606	16	31011183	G	A	0.3583	-0.0183	0.0031	4.86×10^{-09}	0.0367	0.0163	0.02456
rs543874	1	177889480	G	A	0.2667	0.0482	0.0039	2.62×10^{-35}	0.0226	0.0205	0.2696
rs6477694	9	111932342	C	T	0.3583	0.0174	0.0031	2.67×10^{-08}	0.0207	0.0167	0.2159
rs657452	1	49589847	A	G	0.4167	0.0227	0.0031	5.48×10^{-13}	0.0411	0.0164	0.01249
rs6656785	1	75005776	G	A	0.3833	0.0217	0.0031	3.83×10^{-12}	0.0066	0.0161	0.6825
rs6804842	3	25106437	A	G	0.425	-0.0185	0.0031	2.48×10^{-09}	-0.0066	0.0158	0.675
rs7138803	12	50247468	G	A	0.5583	-0.0315	0.0031	8.15×10^{-24}	-0.0024	0.0164	0.8825
rs7141420	14	79899454	T	C	0.6167	0.0235	0.0031	1.23×10^{-14}	-0.024	0.0158	0.129
rs758747	16	3627358	C	T	0.7333	-0.0225	0.0037	7.47×10^{-10}	-0.0196	0.019	0.3017
rs7599312	2	213413231	G	A	0.7083	0.022	0.0034	1.17×10^{-10}	-0.0358	0.0177	0.0434
rs7899106	10	87410904	A	G	0.95	-0.0395	0.0071	2.96×10^{-08}	-0.0256	0.0378	0.4975
rs7903146	10	114758349	T	C	0.25	-0.0234	0.0034	1.11×10^{-11}	-0.0054	0.0173	0.7532
rs879620	16	4015729	C	T	0.4083	-0.0244	0.004	1.06×10^{-09}	0.021	0.0168	0.2106
rs9400239	6	108977663	C	T	0.7	0.0188	0.0033	1.61×10^{-08}	0.0348	0.0169	0.03978
rs1885989	13	28010117	G	A	0.7917	-0.0285	0.0048	2.89×10^{-09}	0.0144	0.0199	0.4679
rs887912	2	59302877	T	C	0.3167	0.0228	0.0034	2.75×10^{-11}	0.0128	0.0173	0.4571
rs2207139	6	50845490	G	A	0.1	0.0447	0.004	4.13×10^{-29}	-0.0001	0.0207	0.9946
rs2287019	19	46202172	C	T	0.85	0.036	0.0042	4.59×10^{-18}	-0.0134	0.0209	0.5209
rs6567160	18	57829135	C	T	0.2833	0.0556	0.0036	3.93×10^{-53}	-0.0354	0.0184	0.05437
rs1421085	16	53800954	C	T	0.45	0.0813	0.0031	8.83×10^{-151}	-0.0005	0.0163	0.9763
rs10840099	11	8667680	C	T	0.725	0.0208	0.0031	3.85×10^{-11}	0.0193	0.0165	0.2411

AD, Alzheimer's disease; BMI, body mass index; BP, base pair position; Chr, chromosome; EA, effect allele; EAF, effect allele frequency; OA, other allele; OR, odd ratios; SE, standard error.

Table B. Instrumental SNPs for WHR and the association between these and AD risk.

SNP	Chr	BP	EA	OA	EAF	beta_WHR	SE_WHR	*P* value_WHR	log-OR_AD	SE_log-OR AD	*P* value_AD
rs1011731	1	170613171	G	A	0.4583	0.019	0.0033	$1.10{\times}10^{-08}$	0.0017	0.0156	0.9154
rs10245353	7	25825139	A	C	0.1833	0.027	0.0042	$1.60{\times}10^{-10}$	-0.0092	0.0208	0.6594
rs10783615	12	52636040	G	A	0.1333	0.035	0.0049	$7.00{\times}10^{-13}$	0.0355	0.0229	0.1219
rs11048470	12	26378550	T	G	0.2333	0.025	0.0037	$6.30{\times}10^{-12}$	-0.0009	0.0175	0.9589
rs1128249	2	165236870	G	T	0.5583	0.021	0.0034	$1.60{\times}10^{-09}$	0.0251	0.0164	0.1249
rs1294421	6	6688148	G	T	0.6	0.025	0.0034	$6.90{\times}10^{-14}$	-0.0144	0.017	0.3976
rs1316952	12	122965503	T	C	0.8833	0.028	0.0049	$7.30{\times}10^{-09}$	-0.0136	0.0229	0.5525
rs1440372	15	64820205	C	T	0.7417	0.021	0.0037	$7.60{\times}10^{-09}$	0.0085	0.0176	0.6275
rs1563355	1	217719724	T	C	0.3136	-0.031	0.0044	$1.70{\times}10^{-12}$	0.0292	0.0168	0.082
rs1569135	2	187823643	A	G	0.5333	0.024	0.0033	$1.00{\times}10^{-12}$	0.0189	0.0153	0.215
rs16996700	20	50415352	T	C	0.7	0.021	0.0037	$1.60{\times}10^{-08}$	0.0238	0.0171	0.1627
rs17451107	3	158280303	T	C	0.625	0.023	0.0035	$3.50{\times}10^{-11}$	-0.0134	0.0161	0.4055
rs2179129	22	27780923	A	G	0.55	0.021	0.0034	$1.20{\times}10^{-09}$	-0.0112	0.0155	0.4694
rs2765539	1	119350941	C	T	0.2917	-0.027	0.0038	$1.10{\times}10^{-12}$	0.0042	0.0171	0.8044
rs2972164	3	12309416	C	T	0.5	0.019	0.0033	$2.40{\times}10^{-08}$	-0.0275	0.0166	0.09678
rs3786897	19	38584848	G	A	0.4083	0.022	0.0034	$4.00{\times}10^{-11}$	-0.0075	0.016	0.6409
rs459193	5	55842508	A	G	0.2167	0.026	0.0038	$6.00{\times}10^{-12}$	0.0007	0.0185	0.9681
rs4640244	17	21224816	G	A	0.375	0.021	0.0037	$3.10{\times}10^{-08}$	0.0204	0.0166	0.2192
rs929641	2	58645881	A	G	0.6167	0.02	0.0033	$4.20{\times}10^{-09}$	-0.0094	0.0156	0.5478
rs9860730	3	64676186	A	G	0.7667	0.023	0.0036	$2.80{\times}10^{-10}$	0.0429	0.0177	0.01525
rs998584	6	43865874	A	C	0.475	0.029	0.0037	$5.00{\times}10^{-15}$	-0.0292	0.0181	0.1061
rs16937664	8	72643014	T	C	0.0583	0.039	0.0064	$1.10{\times}10^{-09}$	0.0104	0.0312	0.7382

SNP	Chr	BP	EA	OA	EAF	beta_WHR	SE_WHR	*P* value_WHR	log-OR_AD	SE_log-OR AD	*P* value_AD
rs7766106	6	127496831	T	C	0.525	0.038	0.0033	7.80×10^{-30}	0.0193	0.0161	0.2301
rs1121980	16	52366748	A	G	0.475	0.043	0.0033	1.30×10^{-38}	0.0005	0.0157	0.9724
rs11663816	18	56027207	C	T	0.3167	0.025	0.0038	2.70×10^{-11}	-0.036	0.0176	0.04071
rs17109256	14	79009746	A	G	0.275	0.023	0.0041	3.00×10^{-08}	0.0042	0.0189	0.8257
rs2287019	19	50894012	C	T	0.85	0.026	0.0045	4.30×10^{-09}	-0.0134	0.0209	0.5209
rs4715213	6	51019050	T	C	0.1	0.024	0.0044	4.00×10^{-08}	0.0028	0.0208	0.8916
rs4929927	11	8615061	G	A	0.725	0.02	0.0034	7.60×10^{-09}	0.0213	0.0165	0.1945

AD, Alzheimer's disease; BP, base pair position; Chr, chromosome; EA, effect allele; EAF, effect allele frequency; OA, other allele; OR, odd ratios; SE, standard error; WHR, waist-to-hip ratio.

Table C. Instrumental SNPs for WC and the association between these and AD risk.

SNP	Chr	BP	EA	OA	EAF	beta_WC	SE_WC	*P* value_WC	log-OR_AD	SE_log-OR AD	*P* value_AD
rs10132280	14	25928179	A	C	0.3333	-0.022	0.0037	2.20×10^{-09}	-0.0053	0.0173	0.7571
rs16894959	6	34825662	C	T	0.1	0.026	0.0048	3.40×10^{-08}	-0.0051	0.022	0.8162
rs16996700	20	50981945	T	C	0.7	0.023	0.0037	1.50×10^{-09}	0.0238	0.0171	0.1627
rs2033529	6	40348653	G	A	0.2583	0.021	0.0037	1.70×10^{-08}	-0.0103	0.0169	0.5434
rs2489623	6	127455821	C	A	0.5583	0.019	0.0034	3.40×10^{-08}	0.0111	0.0161	0.4914
rs2531992	16	4021734	A	G	0.1667	-0.028	0.0048	3.00×10^{-09}	0.0122	0.0217	0.5752
rs3849570	3	81792112	A	C	0.3667	0.021	0.0038	2.20×10^{-08}	0.0319	0.0162	0.04886
rs6163	10	104596924	C	A	0.6083	-0.019	0.0035	3.70×10^{-08}	-0.0012	0.0157	0.9376
rs6440003	3	141094209	G	A	0.5167	-0.021	0.0034	2.90×10^{-10}	0.0199	0.0156	0.2016
rs7239883	18	40147671	G	A	0.3167	0.021	0.0035	2.30×10^{-09}	-0.0339	0.0157	0.03108
rs806794	6	26200677	G	A	0.275	-0.022	0.0037	2.10×10^{-09}	-0.0123	0.0171	0.4737
rs929641	2	58792377	A	G	0.6167	0.021	0.0034	1.20×10^{-09}	-0.0094	0.0156	0.5478
rs10840100	11	8669437	G	A	0.725	0.02	0.0035	5.40×10^{-09}	0.0181	0.017	0.2871
rs10938397	4	45182527	A	G	0.5667	-0.032	0.0035	6.10×10^{-20}	-0.0046	0.016	0.774
rs11165623	1	96893000	A	G	0.4833	0.02	0.0034	5.20×10^{-09}	-0.0033	0.0155	0.8326
rs12429545	13	54102206	G	A	0.9	-0.031	0.0052	2.50×10^{-09}	-0.0077	0.0237	0.7451
rs1516725	3	185824004	T	C	0.0917	-0.031	0.0051	1.70×10^{-09}	-0.0308	0.0227	0.1761
rs17066856	18	58049656	C	T	0.1333	-0.037	0.006	9.00×10^{-10}	0.0172	0.0268	0.5202
rs17381664	1	78048331	C	T	0.425	0.022	0.0035	4.20×10^{-10}	0.0182	0.0161	0.2569
rs2112347	5	75015242	G	T	0.375	-0.025	0.0035	3.20×10^{-13}	0.0208	0.021	0.3241
rs2287019	19	46202172	C	T	0.85	0.035	0.0046	1.70×10^{-14}	-0.0134	0.0209	0.5209
rs2325036	3	85819412	A	C	0.5917	0.023	0.0035	2.10×10^{-11}	0.0024	0.0158	0.8797

SNP	Chr	BP	EA	OA	EAF	beta_WC	SE_WC	*P* value_WC	log-OR_AD	SE_log-OR AD	*P* value_AD
rs2820292	1	201784287	A	C	0.4917	-0.019	0.0034	2.40×10^{-08}	-0.0143	0.0156	0.3611
rs3127553	1	49438005	G	A	0.3667	0.023	0.0035	1.60×10^{-10}	0.0363	0.0168	0.03039
rs633715	1	177852580	C	T	0.2667	0.043	0.0043	3.30×10^{-23}	0.028	0.0205	0.1724
rs6545714	2	59307725	G	A	0.375	0.022	0.0035	1.90×10^{-10}	0.0259	0.0159	0.1037
rs6567160	18	57829135	C	T	0.2833	0.048	0.004	2.60×10^{-33}	-0.0354	0.0184	0.05437
rs6755502	2	635721	T	C	0.125	-0.051	0.0045	2.00×10^{-30}	0.0356	0.02	0.07561
rs7138803	12	50247468	G	A	0.5583	-0.028	0.0035	1.60×10^{-15}	-0.0024	0.0164	0.8825
rs7144011	14	79940383	T	G	0.275	0.033	0.0041	9.40×10^{-16}	0.0029	0.0206	0.8863
rs749671	16	31088347	A	G	0.375	-0.019	0.0035	3.20×10^{-08}	0.0331	0.0161	0.03944
rs7498665	16	28883241	G	A	0.3583	0.034	0.0035	1.40×10^{-22}	-0.0223	0.0162	0.1679
rs7550711	1	110082886	T	C	0.0339	0.058	0.0098	3.40×10^{-09}	0.0009	0.0506	0.9866
rs7903146	10	114758349	T	C	0.25	-0.022	0.0037	3.90×10^{-09}	-0.0054	0.0173	0.7532
rs9400239	6	108977663	C	T	0.7	0.024	0.0036	1.90×10^{-11}	0.0348	0.0169	0.03978
rs943005	6	50865820	T	C	0.1	0.039	0.0044	7.20×10^{-19}	0.0066	0.0213	0.7558
rs7124442	4	27677041	C	T	0.3667	0.03	0.0036	7.20×10^{-17}	0.0185	0.0168	0.2707
rs1421085	16	53800954	C	T	0.45	0.074	0.0035	6.10×10^{-101}	-0.0005	0.0163	0.9763

AD, Alzheimer's disease; BP, base pair position; Chr, chromosome; EA, effect allele; EAF, effect allele frequency; OA, other allele; OR, odd ratios; SE, standard error; WC, waist circumference.

Table D. Instrumental SNPs for WHRadjBMI and the association between these and AD risk.

SNP	Chr	BP	EA	OA	EAF	beta_WHRadjBMI	SE_WHRadjBMI	*P* value_WHRadjBMI	log-OR_AD	SE_log-OR AD	*P* value_AD
rs10245353	7	25858614	A	C	0.1833	0.035	0.0043	8.40×10^{-16}	-0.0092	0.0208	0.6594
rs10804591	3	129334233	A	C	0.85	0.024	0.0042	6.60×10^{-09}	0.0493	0.0186	0.008109
rs10842707	12	26471364	T	C	0.1667	0.032	0.004	4.40×10^{-16}	0.0066	0.0187	0.7256
rs10991437	9	107735920	A	C	0.1	0.031	0.0054	1.00×10^{-08}	-0.0035	0.0244	0.8856
rs11231693	11	63862612	A	G	0.0417	0.041	0.0075	4.50×10^{-08}	0.0107	0.0341	0.7546
rs1128249	2	165528624	G	T	0.5583	0.028	0.0035	2.00×10^{-15}	0.0251	0.0164	0.1249
rs12608504	19	18389135	A	G	0.3417	0.022	0.0036	8.80×10^{-10}	0.0055	0.016	0.7309
rs12679556	8	72514228	G	T	0.2083	0.027	0.004	2.10×10^{-11}	-0.0079	0.0184	0.6663
rs1294410	6	6738752	C	T	0.625	0.031	0.0035	2.00×10^{-18}	-0.0068	0.0162	0.673
rs1385167	2	66200648	G	A	0.1417	0.029	0.0049	1.80×10^{-09}	-0.0473	0.0235	0.04427
rs1440372	15	67033151	C	T	0.7417	0.024	0.0038	1.10×10^{-10}	0.0085	0.0176	0.6275
rs1569135	2	188115398	A	G	0.5333	0.021	0.0034	5.60×10^{-10}	0.0189	0.0153	0.215
rs17451107	3	156797609	T	C	0.625	0.026	0.0036	1.10×10^{-12}	-0.0134	0.0161	0.4055
rs17819328	3	12489342	G	T	0.45	0.021	0.0035	2.40×10^{-09}	-0.0014	0.0164	0.9331
rs1936805	6	127452116	T	C	0.55	0.042	0.0034	3.60×10^{-35}	0.0091	0.0156	0.5586
rs2071449	12	54428011	A	C	0.325	0.028	0.0036	3.00×10^{-14}	0.0078	0.0166	0.6367
rs2294239	22	29449477	A	G	0.55	0.025	0.0035	7.20×10^{-13}	-0.0066	0.0155	0.6694
rs2645294	1	119574587	C	T	0.4655	-0.031	0.0035	1.70×10^{-19}	0.0039	0.0161	0.8087
rs2820443	1	219753509	C	T	0.3	-0.035	0.0037	5.30×10^{-21}	0.0403	0.0178	0.02392
rs303084	4	124066948	A	G	0.7833	0.023	0.0042	3.90×10^{-08}	-0.0129	0.0187	0.4923
rs4081724	19	33824946	G	A	0.85	0.035	0.0051	7.40×10^{-12}	0.0272	0.0224	0.2247
rs459193	5	55806751	A	G	0.2167	0.026	0.0038	1.60×10^{-11}	0.0007	0.0185	0.9681
rs4646404	17	17420199	G	A	0.625	0.027	0.0039	1.40×10^{-11}	-0.004	0.0168	0.8095

SNP	Chr	BP	EA	OA	EAF	beta_WHRadjBMI	SE_WHRadjBMI	*P* value_WHRadjBMI	log-OR_AD	SE_log-OR AD	*P* value_AD
rs4765219	12	124440110	C	A	0.625	0.028	0.0036	1.60×10^{-15}	-0.0081	0.0167	0.6267
rs6090583	20	45558831	A	G	0.4333	0.022	0.0034	6.20×10^{-11}	0.0062	0.0155	0.6877
rs6772129	3	64700425	A	G	0.7583	0.035	0.0037	3.40×10^{-21}	0.0413	0.017	0.01494
rs714515	1	172352990	G	A	0.4583	0.027	0.0034	4.40×10^{-15}	0.002	0.0155	0.8963
rs7705502	5	173320815	A	G	0.2917	0.027	0.0036	4.70×10^{-14}	0.0097	0.017	0.5688
rs8030605	15	56504598	A	G	0.1583	0.03	0.0053	8.80×10^{-09}	0.0181	0.0229	0.4302
rs8042543	15	31708263	C	T	0.85	0.026	0.0043	1.20×10^{-09}	0.0034	0.0202	0.8671
rs878639	20	33894463	A	G	0.6417	0.021	0.0035	5.10×10^{-09}	0.0115	0.0164	0.4848
rs905938	1	154991389	C	T	0.325	-0.025	0.004	7.30×10^{-10}	0.0003	0.0182	0.9887
rs979012	20	6623374	T	C	0.3583	0.027	0.0036	3.30×10^{-14}	0.0497	0.0166	0.002756
rs998584	6	43757896	A	C	0.475	0.043	0.0038	1.10×10^{-29}	-0.0292	0.0181	0.1061
rs9991328	4	89713121	T	C	0.4833	0.018	0.0034	4.50×10^{-08}	0.0066	0.0158	0.6787
rs12139690	1	119491316	A	G	0.1660	-0.024	0.0042	1.20×10^{-08}	0.0012	0.0202	0.9546

AD, Alzheimer's disease; BP, base pair position; Chr, chromosome; EA, effect allele; EAF, effect allele frequency; OA, other allele; OR, odd ratios; SE, standard error; WHRadjBMI, waist-to-ratio adjusted for body mass index.

Supplementary Table 3. The sensitivity analyses of the causal effects amongst instrumental SNPs of each anthropometric trait on the risk of Alzheimer's disease.

Anthropometric trait	Number of IVs	Heterogeneity tests						Horizontal pleiotropy tests					
		Cochran's Q statistic		I^2 statistic	The H statistic		Leave-one-out analysis(outliers)	MR-egger method		MR-PRESSO method (*P* value)			
		Q	*P* _Q		H	95% CI_H (LCL,UCL)		intercept	*P*_intercept	Global test	Outlier test	Distortion test	Outliers removed
BMI	62	100.78007	0.0010	39.47%	1.28	(1.10, 1.50)	rs3817334	0.0090	0.3248	0.0018	rs3817334** (0.0062)	0.7315	-
BMI	61	84.24516	0.0212	28.78%	1.18	(1.01, 1.39)	rs9926784	0.0096	0.2526	0.0211	rs9926784 (0.2318)	-	rs3817334
BMI	60	75.50086	0.0726	21.86%	1.13	(0.96, 1.33)	-	0.0093	0.2446	0.0685	-	-	rs3817334<br>rs9926784
BMI*	65	81.90911	0.0651	21.86%	1.13	(0.97, 1.33)	-	0.0103	0.0899	0.0674	-	-	-
WHR	23	28.76125	0.1518	23.51%	1.14	(0.89, 1.48)	-	-0.0035	0.8669	0.145	-	-	-
WHR*	29	35.20273	0.1640	20.46%	1.12	(0.89, 1.41)	-	0.0010	0.9519	0.1688	-	-	-
WHRadjBMI	36	43.06610	0.1643	18.73%	1.11	(0.90, 1.37)	-	0.0192	0.2310	0.1577	-	-	-
WC	12	14.28680	0.2175	23.01%	1.14	(0.81, 1,59)	-	0.0101	0.8663	0.2052	-	-	-
WC*	41	74.41917	0.0008	46.25%	1.36	(1.14, 1,64)	rs2293576	0.0076	0.4523	0.0009	rs2293576 (0.0082)	0.0981	-
WC*	40	59.46634	0.0189	34.42%	1.23	(1.02, 1.50)	rs10968576	0.0118	0.1995	0.0189	rs10968576 (0.768)	-	rs2293576
WC*	39	54.11927	0.0435	29.78%	1.19	(0.97, 1.46)	rs7531118	0.0103	0.2534	0.0423	rs7531118 (0.936)	-	rs2293576<br>rs10968576
WC*	38	49.26539	0.0855	24.90%	1.15	(0.94, 1.41)	-	0.0115	0.1856	0.0833	-	-	rs2293576<br>rs10968576<br>rs7531118

This chart presents a series of processes of sensitivity analyses of the causal effect of each AT on AD risk. The SNPs in the "Outliers removed" column refer to that these SNPs were identified as outliers based on the results of the tests above, and thus were removed from original IVs when these sensitivity analyses were performed. The heterogeneity test was performed based on the single causal effect for each SNP using Wald ratio method and the overall causal effect for multiple SNPs using inverse-variance weighted (IVW) method. BMI, body mass index; IVs, instrumental variables; LCL/UCL, lower/upper confidence limit; WC, waist circumference; WHR, waist-to-hip ratio; WHRadjBMI, waist-to-hip ratio adjusted for body mass index.

**The sensitivity analyses of additional MR studies.*

***A Phewas of rs3817334 was performed to explore the potential reason, where traits for further evaluation were selected through searching rs3817334 in the NHGRI-EBI GWAS catalog, using a threshold of P<2.04e-05 (0.05/2453 'trait lookups'). We found that fluid intelligence score was associated with rs3817334 (beta=0.0423; $P=3.8\times10^{-8}$), and in further MR analysis of fluid intelligence score and AD, the OR (95% CI) of a 1-SD increase in the fluid intelligence score on the risk of AD was 0.85 (0.77, 0.94). In MR analysis of BMI and AD, the causal estimate only using rs3817334 by Wald ratio method was 0.09 (0.04, 0.22), the direction of which was the same as that of the effect of rs3817334 on AD risk due to fluid intelligence score. These results suggested that for rs3817334 as an IV, BMI was a protective factor of AD risk was due to horizontal pleiotropic effect of fluid intelligence score*

Supplementary Table 4. Summary of results of previous researches about body shape and Alzheimer's disease.

Exposure	Outcome	Study design	Sample size (cases/controls) and source	Association estimated	Adjustment for Confounding factors	References
Higher BMI	AD	A single-sample MR analysis	19692(10079/9613), The Alzheimer's Disease Genetics Consortium (ADGC))	OR (95% CI): 0.95 (0.90, 1.01)	Age, sex, *APOE-ε4*, population stratification	PMID: 26079416
Higher BMI	AD	A single-sample MR analysis	8403, The Health and Retirement Study (HRS)	OR (95% CI): 1.00 (0.75, 1.32)	Age, sex, population stratification	PMID: 26079416
Higher BMI	AD	A single-sample MR analysis	10454(3177/7277), The Genetic and Environmental Risk for AD consortium (GERAD1)	OR (95% CI): 0.96 (0.87, 1.07)	Age, sex	PMID: 26079416
Lower BMI	AD	A follow-up study	95578, Copenhagen General Population Study (CGPS)	HR (95% CI): 1.32 (1.20, 1.46)	Age, sex, hypertension, diabetes, smoking, alcohol intake, physical inactivity, postmenopausal status and hormonal replacement therapy in women, lipid-lowering therapy and educational level	PMID: 28609829
Lower BMI	AD	A Two-sample MR analysis	303958, The Genetic Investigation of Anthropometric Traits (GIANT) (249796) and The International Genomics of Alzheimer's Project (IAGP) (54162(17008/37154))	OR (95% CI): 1.02 (0.86, 1.22)		PMID: 28609829
BMI	AD	A cohort study	388, Subjects recruited at the Seocho Center for Dementia	HR (95% CI): 2.38 (1.17, 4.82)	Age, sex, cognitive intervention, and chronic diseases	PMID: 29719518
BMI	AD	A cohort study	1459(145/1314), A high proportion of Hispanics in northern Manhattan recruited	HR (95% CI): 0.9 (0.7, 1.1)	Ethnicity, education, *APOE-ε4* allele, type 2 diabetes, hypertension, heart disease, non-HDL cholesterol, HDL cholesterol, and stroke	PMID: 21666429
Higher BMI	AD	A cohort study	1836, Japanese Americans living in King County, WA	HR (95% CI): 0.56 (0.33, 0.97)	Age, gender, education, smoking status, alcohol consumption, regular exercise, hypertension, hypercholesterolemia, angina pectoris, diabetes, heart attack, TIA, and stroke	PMID: 19451529

Exposure	Outcome	Study design	Sample size (cases/controls) and source	Association estimated	Adjustment for Confounding factors	References
BMI	AD	A cohort study	893, recipients of 65 years or older residing in northern Manhattan (Washington Heights, Hamilton Heights, Inwood)	HR (95% CI): 0.9 (0.9,1.0)	Age, gender, education, ethnic group, *APOE-ε4*, diabetes, hypertension, LDL, heart disease, stroke, and current smoking in secondary analyses	PMID: 17353383
AD	BMI	A case-control study	301(125/176), ambulatory community-living Chinese older adults recruited from the Memory Clinic of Queen Mary Hospital and social centers for the elderly in Hong Kong.	*P* value=0.002	Dementia diagnostic group, hypertension, diabetes mellitus (DM), previous stroke, arthritis, apolipoprotein E genotype, and systolic blood pressure (BP)	PMID: 19221407
Underweight (BMI<20)	AD	A case-control study	125(50/75), Participants from Launceston, Australia, and Bristol, England	OR (95% CI): 7.9 (1.0, 66.3)	Age, sex, location, systolic blood pressure, cholesterol and glucose	PMID: 16847377
Overweight (BMI: 25-29.9)	AD	A case-control study	125(50/75), Participants from Launceston, Australia, and Bristol, England	OR (95% CI): 1.8 (0.7, 5.1)	Age, sex, location, systolic blood pressure, cholesterol and glucose	PMID: 16847377
Obese (BMI≥30)	AD	A case-control study	125(50/75), Participants from Launceston, Australia, and Bristol, England	OR (95% CI): 12.6 (2.8, 56.5)	Age, sex, location, systolic blood pressure, cholesterol and glucose	PMID: 16847377
Obese (BMI≥30)	AD	A meta-analysis of cohort studied	United States	RR (95% CI): 1.98 (1.24,3.14)		PMID: 23401370
BMI	AD	A meta-analysis based on studies included in prior systematic reviews	(Cochrane Database of Systematic Reviews (all years) and PUBMED (2005–2011) to identify English-language systematic reviews and meta-analyses for associations with AD or dementia.)	RR (95% CI): 1.60 (1.34,1.92)		PMID: 28259165
WHR	AD	A cohort study	1459(145/1314), A high proportion of Hispanics in northern Manhattan recruited	HR (95% CI): 1.4 (1.1, 1.8)	Ethnicity, education, *APOE-ε4* allele, type 2 diabetes, hypertension, heart disease, non-HDL cholesterol, HDL cholesterol, and stroke	PMID: 21666429

**Exposure**	**Outcome**	**Study design**	**Sample size (cases/controls) and source**	**Association estimated**	**Adjustment for Confounding factors**	**References**
WHR	AD	A cohort study	1051, Participants of aged 65 years or older residing in northern Manhattan, New York	HR (95% CI):<br>1.13 (1.09, 1.16)	age, sex, diabetes, hypertension, current smoking, HDL-C levels, education, ethnicity, and *APOE-ε4* genotype	PMID: 20625090
High WHR:<br>>0.8(women),<br>>0.9 (men)	AD	A case-control study	125(50/75), Participants from Launceston, Australia, and Bristol, England	OR (95% CI):<br>2.0 (0.8, 5.0)	Age, sex, location, systolic blood pressure, cholesterol and glucose	PMID: 16847377
WC	AD	A cohort study	1459(145/1314), A high proportion of Hispanics in northern Manhattan recruited	HR (95% CI):<br>0.9 (0.8, 1.2)	Ethnicity, education, *APOE-ε4* allele, type 2 diabetes, hypertension, heart disease, non-HDL cholesterol, HDL cholesterol, and stroke	PMID: 21666429
WC	AD	A cohort study	907, recipients of 65 years or older residing in northern Manhattan (Washington Heights, Hamilton Heights, Inwood)	HR (95% CI):<br>1.0 (0.9,1.0)	Age, gender, education, ethnic group, *APOE-ε4*, diabetes, hypertension, LDL, heart disease, stroke, and current smoking in secondary analyses	PMID: 17353383
AD	WC	A case-control study	301(125/176), ambulatory community-living Chinese older adults recruited from the Memory Clinic of Queen Mary Hospital and social centers for the elderly in Hong Kong	*P* value=0.018	Dementia diagnostic group, hypertension, diabetes mellitus (DM), previous stroke, arthritis, apolipoprotein E genotype, and systolic blood pressure (BP)	PMID: 19221407

BMI, body mass index; WHR, waist-to-hip ratio; WC, waist circumference; AD, Alzheimer's disease; MR, Mendelian randomization.
